# Discovery and validation of cell cycle arrest biomarkers in human acute kidney injury

**DOI:** 10.1186/cc12503

**Published:** 2013-02-06

**Authors:** Kianoush Kashani, Ali Al-Khafaji, Thomas Ardiles, Antonio Artigas, Sean M Bagshaw, Max Bell, Azra Bihorac, Robert Birkhahn, Cynthia M Cely, Lakhmir S Chawla, Danielle L Davison, Thorsten Feldkamp, Lui G Forni, Michelle Ng Gong, Kyle J Gunnerson, Michael Haase, James Hackett, Patrick M Honore, Eric AJ Hoste, Olivier Joannes-Boyau, Michael Joannidis, Patrick Kim, Jay L Koyner, Daniel T Laskowitz, Matthew E Lissauer, Gernot Marx, Peter A McCullough, Scott Mullaney, Marlies Ostermann, Thomas Rimmelé, Nathan I Shapiro, Andrew D Shaw, Jing Shi, Amy M Sprague, Jean-Louis Vincent, Christophe Vinsonneau, Ludwig Wagner, Michael G Walker, R Gentry Wilkerson, Kai Zacharowski, John A Kellum

**Affiliations:** 1 Division of Pulmonary and Critical Care Medicine, Mayo Clinic, 200 First Street SW, Rochester, MN 55905, USA; 2 Department of Critical Care Medicine, University of Pittsburgh School of Medicine, 3550 Terrace Street, Pittsburgh, PA 15213, USA; 3 Department of Critical Care, Maricopa Integrated Health System, 2601 E Roosevelt Street, Phoenix, AZ 85008, USA; 4 Critical Care Center, Sabadell Hospital, CIBER Enfermedades Respiratorias, Autonomous University of Barcelona, Parc Tauli s/n, Sabadell, Barcelona 8208, Spain; 5 Division of Critical Care Medicine, Faculty of Medicine and Dentistry, University of Alberta, 3C1.12 Walter C. Mackenzie Centre, 8440 112 Street NW, Edmonton, Alberta T6G 2B7, Canada; 6 Department of Anesthesia and Intensive Care Medicine, Karolinska University Hospital, Karolinskavagen, Solna, Stockholm SE-171 76, Sweden; 7 Department of Anesthesiology, University of Florida, 1660 SW Archer Road, Gainesville, FL 32611, USA; 8 Department of Emergency Medicine, New York Methodist Hospital, 506 6th Street, Brooklyn, NY 11215, USA; 9 Bruce W. Carter Department of Veterans Affairs Medical Center, 1201 NW 16th Street, Miami, FL 33125, USA; 10 Department of Anesthesiology and Critical Care Medicine, George Washington University Medical Center, 900 23rd Street NW, Washington, DC 20037, USA; 11 Department of Nephrology, University Hospital Essen, University Duisburg-Essen, Hufelandstrasse 55, Essen, 45147, Germany; 12 Intensive Care Medicine, Western Sussex Hospitals Trust, Lyndhurst Road, Worthing, West Sussex, BN11 2DH, UK; 13 Department of Medicine, Montefiore Medical Center, Albert Einstein College of Medicine, 111 East 210th Street, Bronx, NY 10467, USA; 14 Departments of Anesthesiology and Emergency Medicine, Virginia Commonwealth University Medical Center, 1200 East Broad Street, Richmond, VA 23298, USA; 15 Department of Nephrology, Otto-von-Guericke-Universitat Magdeburg, Leipziger Strasse 44, Magdeburg, 39120, Germany; 16 Hackett & Associates, Inc., 14419 Rancho Del Prado Trail, San Diego, CA 92127, USA; 17 ICU Department, Universitair Ziekenhuis Brussel (UZB), Vrije Universiteit Brussel (VUB), Laarbeeklaan 101, Brussels 1090, Belgium; 18 Intensive Care Unit, Ghent University Hospital, De Pintelaan 185, Ghent, 9000, Belgium; 19 Anaesthesiology and Critical Care Department 2, University Hospital of Bordeaux, 1 Avenue De Magellon, Pessac, 33600, France; 20 Department of Internal Medicine, ICU, Medical University Innsbruck, Anichstrasse 35, Innsbruck, A-6020, Austria; 21 Traumatology, Surgical Critical Care and Emergency Surgery, Hospital of the University of Pennsylvania, 3400 Spruce Street, Philadelphia, PA 19104, USA; 22 Department of Medicine, University of Chicago, 6030 South Ellis Avenue, Chicago, IL 60637, USA; 23 Department of Medicine, Duke University Medical Center, 2301 Erwin Road, Durham, NC 27710, USA; 24 Department of Surgery, University of Maryland School of Medicine, 22 South Greene Street, Baltimore, MD 21201, USA; 25 Department of Intensive Care, Universitätsklinikum der RWTH Aachen, Pauwelsstrasse 30, Aachen, 52074, Germany; 26 Department of Medicine, St John Providence Health System, Providence Hospitals and Medical Centers, Providence Park Heart Institute, 47601 Grand River Avenue, Novi, MI 48374, USA; 27 Department of Medicine, University of California San Diego, 200 West Arbor Drive, San Diego, CA 92103, USA; 28 Department of Critical Care, King's College London, Guy's and St Thomas' Hospital, Westminster Bridge Road, London, SE1 7EH, UK; 29 Service D'Anesthésie Réanimation, Edouard Herriot Hospital, Hospices civils de Lyon, 5 Place d'Arsonval, Lyon, 69003, France; 30 Department of Emergency Medicine, Beth Israel Deaconess Medical Center, 1 Deaconess Road, Boston, MA 2215, USA; 31 Department of Anesthesia, Duke University Medical Center/Durham Veterans Affairs Medical Center, 508 Fulton Street, Durham, NC 27705, USA; 32 Walker Biosciences, 6321 Allston Street, Carlsbad, CA 92009, USA; 33 Department of Medicine, Joseph M. Still Research Foundation, 3675 J. Dewey Gray Circle, Augusta, GA 30909, USA; 34 Department of Intensive Care, Erasme University Hospital, Route De Lennik 808, Brussels, 1070, Belgium; 35 Department of Intensive Care, Hospital Marc Jacquet, 2 Rue Freteau De Peny, Melun, 77011, France; 36 Department of Internal Medicine, Medical University of Vienna, Spitalgasse 23, Vienna 1090, Austria; 37 Department of Emergency Medicine, Tampa General Hospital, 1 Davis Boulevard, Tampa, FL 33606, USA; 38 Clinic of Anesthesiology, Intensive Care Medicine and Pain Therapy, University Hospital Frankfurt, Theodor-Stern-Kai 7, Frankfurt am Main, 60590, Germany; 39 Department of Critical Care Medicine, University of Pittsburgh, School of Medicine, 3550 Terrace Street, Pittsburgh, PA 15213, USA

## Abstract

**Introduction:**

Acute kidney injury (AKI) can evolve quickly and clinical measures of function often fail to detect AKI at a time when interventions are likely to provide benefit. Identifying early markers of kidney damage has been difficult due to the complex nature of human AKI, in which multiple etiologies exist. The objective of this study was to identify and validate novel biomarkers of AKI.

**Methods:**

We performed two multicenter observational studies in critically ill patients at risk for AKI - discovery and validation. The top two markers from discovery were validated in a second study (Sapphire) and compared to a number of previously described biomarkers. In the discovery phase, we enrolled 522 adults in three distinct cohorts including patients with sepsis, shock, major surgery, and trauma and examined over 300 markers. In the Sapphire validation study, we enrolled 744 adult subjects with critical illness and without evidence of AKI at enrollment; the final analysis cohort was a heterogeneous sample of 728 critically ill patients. The primary endpoint was moderate to severe AKI (KDIGO stage 2 to 3) within 12 hours of sample collection.

**Results:**

Moderate to severe AKI occurred in 14% of Sapphire subjects. The two top biomarkers from discovery were validated. Urine insulin-like growth factor-binding protein 7 (IGFBP7) and tissue inhibitor of metalloproteinases-2 (TIMP-2), both inducers of G_1 _cell cycle arrest, a key mechanism implicated in AKI, together demonstrated an AUC of 0.80 (0.76 and 0.79 alone). Urine [TIMP-2]*·*[IGFBP7] was significantly superior to all previously described markers of AKI (*P *<0.002), none of which achieved an AUC >0.72. Furthermore, [TIMP-2]*·*[IGFBP7] significantly improved risk stratification when added to a nine-variable clinical model when analyzed using Cox proportional hazards model, generalized estimating equation, integrated discrimination improvement or net reclassification improvement. Finally, in sensitivity analyses [TIMP-2]*·*[IGFBP7] remained significant and superior to all other markers regardless of changes in reference creatinine method.

**Conclusions:**

Two novel markers for AKI have been identified and validated in independent multicenter cohorts. Both markers are superior to existing markers, provide additional information over clinical variables and add mechanistic insight into AKI.

**Trial registration:**

ClinicalTrials.gov number NCT01209169.

## Introduction

Acute kidney injury (AKI) is a vexing clinical problem, in part, because it is difficult to identify before there is loss of organ function, which may then become irreversible [1]. Patients developing AKI have a markedly increased risk of death prior to hospital discharge [2,3] and survivors also appear to be at significant short- and long-term risk for complications [4,5]. Available therapies are mainly predicated on supportive measures and the removal of nephrotoxic agents [6]. Thus, risk assessment for AKI is recommended by clinical practice guidelines [6]. However, risk stratification remains very difficult, mainly due to limited sensitivity and specificity of the available diagnostic tests for AKI [7]. Prior efforts at identifying biomarkers for AKI have been hampered by the heterogeneous nature of the condition. Many different etiologies for AKI have been reported (for example sepsis, nephrotoxins, ischemia), and in any given patient the cause is typically thought to be multifactorial [8]. Here we report the results of a prospective, multicenter investigation in which two novel biomarkers for AKI were identified in a discovery cohort of critically ill adult patients and subsequently validated using a clinical assay and compared to existing markers of AKI in an independent validation cohort of heterogeneous critically ill patients.

## Materials and methods

### Subjects

**Figure 1:**
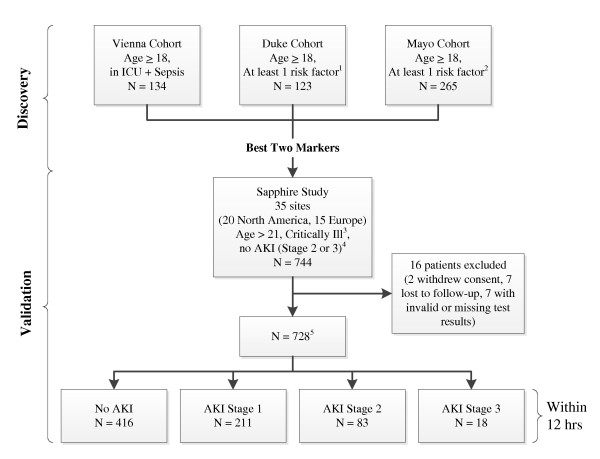
**Study design and number of patients in cohorts**. ^1^Risk factors included sepsis, hypotension, major trauma, hemorrhage, radiocontrast exposure, or major surgery or requirement for ICU admission. All enrolled patients were in the ICU. ^2^Risk factors included hypotension, sepsis, IV antibiotics, radiocontrast exposure, increased intra-abdominal pressure with acute decompensated heart failure, or severe trauma as the primary reason for ICU admission and likely to be in the ICU for 48 hours. ^3^Critical illness was defined as admission to an ICU and sepsis-related organ failure assessment (SOFA) score [32] ≥2 for respiratory or ≥1 for cardiovascular. ^4^Initially patients with acute kidney injury (AKI) stage 1 were also excluded but this was changed at the first protocol amendment. ^5^A total of 728 patients had test results for urinary biomarkers. A total of 726 patients had test results for plasma biomarkers.

We conducted a two-stage program in which we first collected blood and urine samples from three distinct cohorts (Discovery study) to identify novel protein biomarkers for AKI. These single-center studies were used to identify the best biomarkers among 340 proteins, including novel candidates and previously described biomarkers such as kidney injury marker-1 (KIM-1), neutrophil gelatinase-associated lipocalin (NGAL), cystatin-C, interleukin-18 (IL-18), pi-glutathione S-transferase (pi-GST), and liver fatty acid-binding protein (L-FABP). Data from all three cohorts were pooled for analysis. A fourth cohort (Sapphire study) was assembled from 35 clinical sites in North America and Europe and used to validate the performance of the best biomarkers (urine tissue inhibitor of metalloproteinases-2 (TIMP-2) and insulin-like growth factor-binding protein 7 (IGFBP7)) from the Discovery study (Figure 1). The Sapphire study was approved by the Western Institutional Review Board (Olympia, Washington, USA). In addition, the study protocols were approved by investigational review boards/ethics committees as required, by each participating institution. All subjects (or authorized representatives) provided written informed consent.

The Sapphire study was designed and reported according to the STROBE guidelines [9]. As shown in Figure 1, the Discovery study enrolled patients who were admitted to an intensive care unit (any type), were at least 18 years of age and typically had at least one recognized risk factor for AKI. The Sapphire (validation) study enrolled critically ill patients who were at least 21 years of age, admitted to the intensive care unit within 24 hours of enrollment, expected to remain in the ICU with a urinary catheter for at least 48 hours and were critically ill (respiratory or cardiovascular dysfunction). Patients with known existing moderate or severe AKI (KDIGO [6] stage 2 or 3) were excluded. Sample size for the Sapphire study was based on the results of the Discovery study and is explained in detail in Additional file 1.

### Sample and data collection

Paired urine and blood samples were collected at enrollment and up to 18 hours later by standard methods and centrifuged. Plasma (EDTA), serum and urine supernatants were frozen, shipped on dry ice, stored at ≤-70°C and thawed immediately prior to analysis. Clinical data including patient demographics, prior health history, serum creatinine, and hourly urine output as available in the hospital record were collected. Samples were analyzed at Astute Medical by technicians blinded to clinical data. Password-protected, anonymized clinical data collected with electronic case-report forms resided on servers at independent sites (Acumen Healthcare Solutions, Plymouth, MN, USA and Medidata Solutions, New York, NY, USA for Discovery and Sapphire studies, respectively).

### Clinical endpoints

AKI status was classified using the RIFLE [10] or AKIN criteria [11] together as described in the recent KDIGO international guideline [6] based on the serum creatinine (sCR) and urine output (UO) available in the hospital record. The primary endpoint for the Sapphire study was the development of moderate or severe AKI (KDIGO stage 2 or 3) within 12 hours of sample collection. The reference values for serum creatinine were obtained as follows: if at least five values were available the median of all values available from six months to six days prior to enrollment was used. Otherwise, the lowest value in the five days prior to enrollment was used. If no pre-enrollment creatinine was available, the creatinine value at the time of enrollment was used (see Additional file 1 for full details). We performed sensitivity analyses by repeating the primary analysis using several different methods of reference creatinine assignment. Details including the sensitivity analyses are given in Additional file 1. Secondary endpoints for the purpose of characterizing the patient population included renal replacement therapy at any time during hospitalization, survival and major adverse kidney events. We defined major adverse kidney events (MAKE_30_) as the composite of death, use of renal replacement therapy, or persistence of renal dysfunction (defined by serum creatinine ≥200% of reference) at hospital discharge truncated at 30 days [12].

### Biomarker selection

Candidate biomarkers were identified through hypotheses based on AKI pathophysiology. Medline was searched from March 1995 to January 2011 for full reports of original research and review articles with the terms 'Acute kidney injury' OR 'Acute renal failure' AND/OR including one or more of the following terms: inflammation, apoptosis, necrosis, endothelial injury, cell-cell and cell-matrix adhesion, cytoprotection, oxidative processes and cell cycle. Abstracts were downloaded for all titles of potential relevance. Full papers were downloaded when the abstract was deemed relevant. A total of 340 candidate biomarkers were identified for analysis in the Discovery study. Proteins expressed in the kidney and peripherally (for example, in leukocytes) were included in the analyses. Biomarkers were ranked by ability to predict development of AKI RIFLE I or F within 12 to 36 hours. All possible combinations of two to four biomarkers (novel or previously described) were ranked to ensure that any biomarker that might contribute in top-performing combinations of biomarkers was retained.

### Laboratory methods

Biomarkers were measured with single or multiplexed immunoassays using standard ELISA, Luminex 200 (Luminex, Austin, TX, USA), MSD SECTOR Imager 6000 (Meso Scale Discovery, Gaithersburg, MD, USA), or Astute140™ Meter (Astute Medical, San Diego, CA, USA) platforms. Immunoassays were either developed by Astute Medical or obtained from vendors and used as recommended by the vendor or modified to optimize performance. Novel biomarkers were measured with research assays (TIMP-2: R&D Systems, Minneapolis, MN, USA; IGFBP7: Millipore, Billerica, MA, USA) in the Discovery study and with the NephroCheck™ Test (Astute Medical, San Diego, CA, USA) in the Sapphire study. The NephroCheck Test was developed to simultaneously measure the two top-performing biomarkers (urine [TIMP-2]*·*[IGFBP7]) from the Discovery study using a platform that can be used clinically. Previously described biomarkers of AKI (including urine KIM-1, urine and plasma NGAL, plasma cystatin-C, urine IL-18, urine pi-GST, and urine L-FABP) were measured with commercially available assays (see Additional file 1).

### Statistical analysis

The primary analysis was based on area under the receiver-operating characteristics curve (AUC) comparing [TIMP-2]*·*[IGFBP7] to previously described biomarkers for the development of the primary endpoint (KDIGO stage 2 to 3 within 12 hours of sample collection, for samples collected within 18 hours of enrollment). We also characterized the distributions of [TIMP-2]*·*[IGFBP7] values and several existing marker levels for AKI by severity and for various non-AKI conditions. We characterized risk for KDIGO stage 2 to 3 within 12 hours of sample collection and for MAKE_30 _by [TIMP-2]*·*[IGFBP7]. We calculated relative risk for KDIGO stage 2 to 3 by tertile. We computed the AUCs for novel and existing biomarkers in several subgroups of patients (see Additional file 1). We constructed a model based on the clinical variables found to be associated with the primary endpoint (*P *<0.1) and examined whether the addition of [TIMP-2]*·*[IGFBP7] improved risk prediction using time to event, integrated discrimination improvement (IDI), category-free net reclassification improvement (cfNRI) and risk assessment plot analyses (see Additional file 1). Statistical analyses and biomarker selection in the Discovery study were performed by Astute Medical. The primary statistical analyses for the Sapphire study were performed by a team of independent statisticians (MW, JS, and JH). Statistical analyses were performed using SAS 9.3 (SAS Institute, Cary, NC, USA) and R 2.12 [13]. For all analyses, two-sided *P *values less than 0.05 were considered statistically significant. Categorical variables were analyzed using the Fisher exact test or logistic regression. AUC was calculated as empirical AUC with bootstrap confidence intervals to handle subjects with more than one sample collected within 18 hours of enrollment. Differences between AUCs were tested using bootstrap sampling. Time to event analyses used Cox proportional hazards regression with the log transform of [TIMP-2]*·*[IGFBP7] because the distribution was right-skewed. Tests of trend in relative risk across tertiles used the Jonckheere-Terpstra test [14].

## Results

### Subject characteristics and event rates

We enrolled 744 subjects in the validation cohort, 460 (62%) from North American sites and 284 (38%) from Europe (Figure 1). Sixteen patients (2%) were excluded from the analysis cohort because of withdrawal of consent, loss to follow-up, or invalid or missing test results leaving 728 subjects for the analysis. Demographic information for the analysis cohort is depicted in Table 1. Overall, 101 subjects (14%) in the analysis cohort met the primary endpoint of moderate or severe AKI (11% stage 2, 2.5% stage 3) within 12 hours. In addition, 218 (30%) developed AKI within seven days (22% stage 2, 8% stage 3) and 49 (6.7%) underwent renal replacement therapy during the hospital stay truncated at 30 days. A total of 121 (17%) died prior to hospital discharge truncated at 30 days. Finally, 161 subjects (22%) met the MAKE_30 _endpoint.

**Table 1:** Baseline characteristics for Sapphire study patients.

		Endpoint positive	Endpoint negative	All patients	*P *values
**All patients**		101	627	728	

**Male**		65 (64%)	384 (61%)	449 (62%)	0.58

**Age^1^**		65 (57-77)	64 (52-73)	64 (53-73)	0.048

**Race**					0.98
	**White**	81 (80%)	492 (78%)	573 (79%)	
	**Black**	11 (11%)	76 (12%)	87 (12%)	
	**Other/Unknown**	9 (9%)	59 (9%)	68 (9%)	

**Chronic comorbidities**					
	**Chronic kidney disease**	14 (14%)	51 (8%)	65 (9%)	0.14
	**Diabetes mellitus**	39 (39%)	171 (27%)	210 (29%)	0.064
	**Congestive heart failure**	23 (23%)	99 (16%)	122 (17%)	0.17
	**Coronary artery disease**	33 (33%)	187 (30%)	220 (30%)	0.48
	**Hypertension**	76 (75%)	357 (57%)	433 (59%)	0.001
	**Chronic obstructive pulmonary disease**	21 (21%)	141 (22%)	162 (22%)	0.80
	**Cancer**	25 (25%)	163 (26%)	188 (26%)	0.53

**ICU type**					0.47
	**Medical**	40 (40%)	185 (30%)	225 (31%)	
	**Surgical**	24 (24%)	155 (25%)	179 (25%)	
	**Combined ICU**	14 (14%)	133 (21%)	147 (20%)	
	**Cardiac surgery**	6 (6%)	55 (9%)	61 (8%)	
	**Neurologic**	5 (5%)	34 (5%)	39 (5%)	
	**Coronary care unit**	5 (5%)	25 (4%)	30 (4%)	
	**Trauma**	4 (4%)	20 (3%)	24 (3%)	
	**Other/Unknown**	3 (3%)	20 (3%)	23 (3%)	

**Reason for ICU admission^2^**					
	**Respiratory**	47 (47%)	263 (42%)	310 (43%)	0.39
	**Surgery**	32 (32%)	215 (34%)	247 (34%)	0.65
	**Cardiovascular**	41 (41%)	202 (32%)	243 (33%)	0.11
	**Sepsis**	26 (26%)	110 (18%)	136 (19%)	0.055
	**Neurological**	8 (8%)	62 (10%)	70 (10%)	0.72
	**Trauma**	4 (4%)	51 (8%)	55 (8%)	0.16
	**Other**	21 (21%)	105 (17%)	126 (17%)	0.32

**Enrollment serum creatinine^1,3^**		1.4 (0.9-1.8)	0.9 (0.7-1.2)	0.9 (0.7-1.2)	<0.001

**APACHE III^1,4^**		85 (59-106)	67 (51-88)	69 (51-91)	<0.001

Baseline characteristics are shown for all patients in the study and patients that are either negative or positive for the primary study endpoint (KDIGO stage 2 or 3 within 12 hours). ^1^Median (interquartile range); ^2^percentages for reason for ICU admission do not sum to 100% because more than one reason can be given; ^3^value in hospital record closest to enrollment time; ^4 ^calculated from source data by the study sponsor. APACHE III, Acute Physiology and Chronic Health Evaluation III.

### Novel biomarker performance

**Figure 2:**
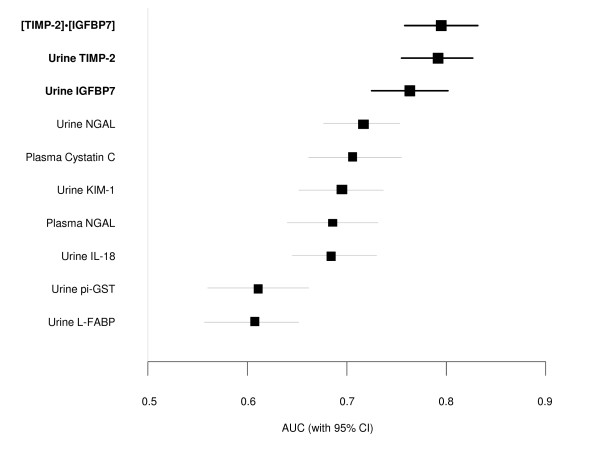
**Area under the receiver-operating characteristics curve (AUC) for novel urinary biomarkers and existing biomarkers of acute kidney injury for the primary Sapphire study endpoint (KDIGO stage 2 or 3 within 12 hours of sample collection)**. Samples were collected within 18 hours of enrollment. The AUC for urinary [TIMP-2]·[IGFBP7] is larger than for the existing biomarkers (*P *value <0.002). IGFBP7, insulin-like growth factor-binding protein 7; IL-18, interleukin-18; KIM-1, kidney injury marker-1; L-FABP, liver fatty acid-binding protein; NGAL, neutrophil gelatinase-associated lipocalin; pi-GST, pi-Glutathione S-transferase; TIMP-2, tissue inhibitor of metalloproteinases-2.

Urinary insulin-like growth factor binding protein (IGFBP) 7 and tissue inhibitor of metalloproteinase (TIMP)-2 were the best-performing markers in the discovery study (AUC = 0.77 and 0.75, respectively, for RIFLE-I/F within 12 to 36 hours; Table S8 in Additional file 1), and were therefore the markers we sought to validate in the Sapphire study. Because these markers appeared to have additive predictive value when used together in the discovery cohort, we made the decision to use the combination (a simple two-marker panel) as the primary readout for the validation. In order to ensure that this readout could be interpreted using a commercial assay platform, we used the NephroCheck Test for the Sapphire study. The test result is a simple multiplication of the two markers ([TIMP-2]*·*[IGFBP7]) (see Additional file 1). In the Sapphire study, [TIMP-2]*·*[IGFBP7] exhibited an AUC of 0.80 for development of AKI (stage 2 or 3) within 12 hours and alone IGFBP7 and TIMP-2 each exhibited an AUC of 0.76 and 0.79 respectively (Figure 2 and Table S1 in Additional file 1).

### Comparison of biomarker performance to previously described AKI biomarkers

**Figure 3:**
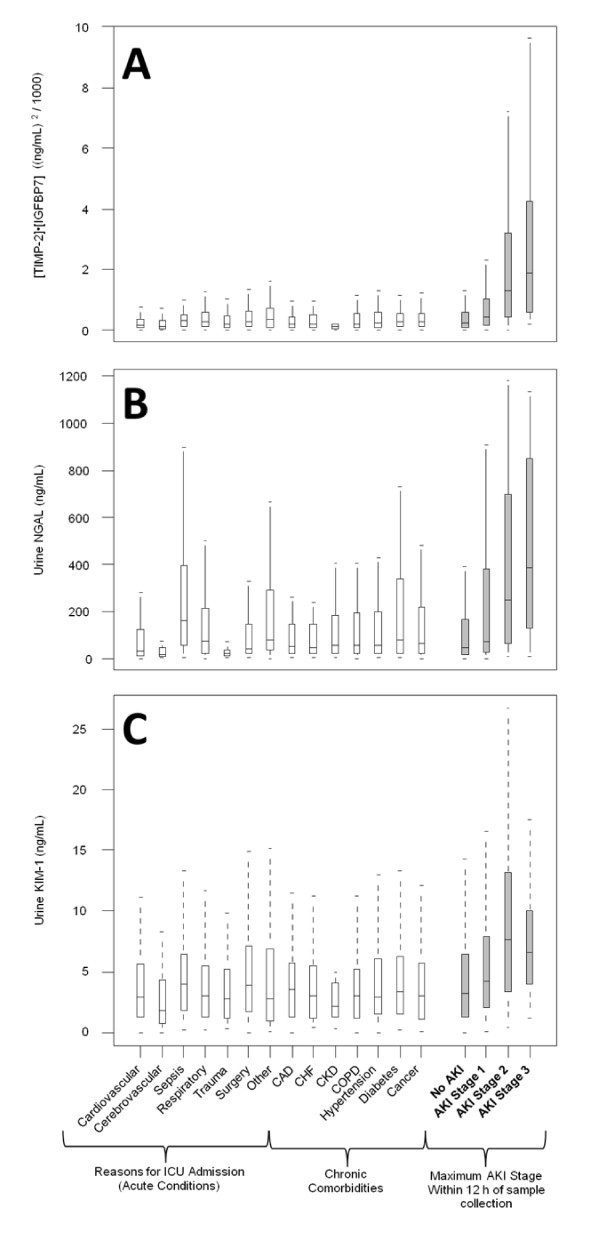
**Discrimination between non-AKI conditions and AKI of different severities for (A) urine [TIMP-2]·[IGFBP7], (B) urine NGAL, and (C) urine KIM-1**. Open boxes represent Sapphire subjects who did not have AKI (of any stage) within seven days. Shaded boxes represent Sapphire subjects stratified by maximum AKI stage within 12 hours of sample collection. Boxes and whiskers show interquartile ranges and total observed ranges (censored by 1.5 times the box range), respectively. Samples were collected within 18 hours of enrollment. AKI, acute kidney injury; IGFBP7, insulin-like growth factor-binding protein 7; KIM-1, kidney injury marker-1; NGAL, neutrophil gelatinase-associated lipocalin; TIMP-2, tissue inhibitor of metalloproteinases-2.

Figure 2 also shows the AUCs for several previously described AKI biomarkers (urine and plasma NGAL, plasma cystatin-C, and KIM-1, IL-18, pi-GST, and L-FABP in the urine). The AUC for urine [TIMP-2]*·*[IGFBP7] was significantly greater (*P *<0.002) than any of these existing biomarkers. We also examined the performance of urine [TIMP-2]*·*[IGFBP7] compared to various other markers including urine KIM-1 and urine NGAL in terms of discrimination between AKI of different severities and various non-AKI conditions including chronic kidney disease (Figure 3). Unlike existing markers, [TIMP-2]·[IGFBP7] showed clear separation between AKI and non-AKI conditions.

### Risk of AKI and MAKE_30 _by [TIMP-2]·[IGFBP7] result

**Figure 4:**
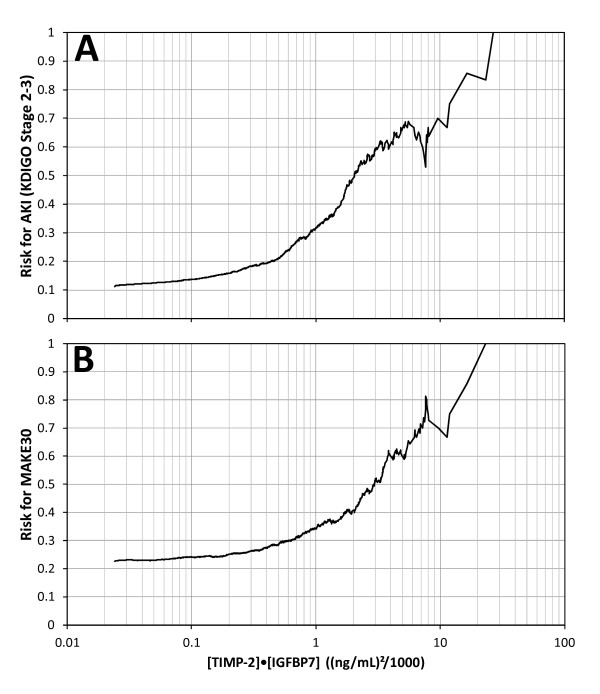
**Risk for KDIGO stage 2 to 3 AKI (A) and MAKE_30 _(B) as a function of urine [TIMP-2]·[IGFBP7]**. Risk at each [TIMP-2]·[IGFBP7] value along the abscissa was calculated as follows: the number of samples positive for the endpoint that had [TIMP-2]·[IGFBP7] above the abscissa value divided by the total number of samples that had [TIMP-2]·[IGFBP7] above the abscissa value. Slightly more than 50% of the samples had a [TIMP-2]·[IGFBP7] value above 0.3 where risk began to elevate sharply and about 10% of the samples had a [TIMP-2]·[IGFBP7] value above 2.0 where risk almost doubled and quintupled for MAKE**_30 _**and AKI, respectively. AKI, acute kidney injury; IGFBP7, insulin-like growth factor-binding protein 7; TIMP-2, tissue inhibitor of metalloproteinases-2.

Risk of AKI (KDIGO stage 2 to 3 within 12 hours) and MAKE_30 _elevated sharply for [TIMP-2]·[IGFBP7] above 0.3 and almost quintupled and doubled, respectively, for [TIMP-2]·[IGFBP7] above 2.0 (Figure 4). Relative risk for AKI (KDIGO 2 to 3 within 12 hours) was also examined by tertile (data not shown). Compared to the lowest tertile, subjects with a result in the middle tertile had a 3-fold relative risk (*P *<0.001) and those in the highest tertile had a nearly 10-fold relative risk (*P *<0.001).

### Additional information from biomarkers over clinical variables

We also examined whether [TIMP-2]*·*[IGFBP7] enhances predictive ability over clinical variables. [TIMP-2]*·*[IGFBP7] significantly improved risk prediction when added to a nine-parameter clinical model (including serum creatinine at matched time points with biomarkers) for the primary endpoint, using time to event, IDI, cfNRI and risk assessment plot analyses (Tables S4-S6 in Additional file 1 and Figure S3 in Additional file 1). All analyses showed significant enhancement by the addition of [TIMP-2]*·*[IGFBP7] with [TIMP-2]*·*[IGFBP7] remaining strongly associated with AKI in all models.

### Sensitivity analyses

Finally, we performed a variety of sensitivity analyses (Table S7 in Additional file 1). We examined several methods of assigning the serum creatinine reference value. We also examined the effect of including or excluding patients who had (unbeknownst to the investigators at the time) reached the endpoint prior to sample collection and including only the enrollment sample (see Additional file 1). For all sensitivity analyses, our conclusions were unchanged and the [TIMP-2]·[IGFBP7] AUC was not different from the primary analysis (point estimate for AUC within the 95% confidence interval) and was higher than the AUC of all previously described biomarkers tested.

## Discussion

To our knowledge this is the first report of an AKI biomarker study that used a development-validation approach with separate patient cohorts in the context of a large prospective multicenter trial framework. Our results are striking not only in terms of identifying new robust markers that have improved performance characteristics when directly compared with existing methods for detecting risk for AKI, but also provide significant additional information over clinical data as evidenced by IDI, cfNRI and Cox models. Furthermore, these molecules are known to be associated with mechanisms recently implicated in the pathogenesis of AKI [15-17]. Thus, our results are important on two levels, development of new diagnostics and bolstering understanding of the mechanism of disease.

AKI poses both unique opportunities and challenges for development of biomarkers to aid in risk assessment. The ability to sample fluid 'proximal' to the site of injury, (that is urine), is an important advantage. However, AKI is also challenging because traditional methods of biomarker discovery often rely on model systems where pathogenesis is well understood or on tissues taken from patients with disease [18] and, since biopsies are rarely obtained from patients with AKI, these tissues are not easy to obtain. Further challenges exist because AKI is not a single disease but a complex syndrome with multiple underlying etiologies [6,19]. Animal models are usually the source of tissue for many biomarker discovery programs, but these rarely, if ever, exemplify the full complexity of human AKI [20]. For these reasons, we chose to discover potential biomarkers in critically ill humans with and without AKI as opposed to relying on animal models. This approach has the distinct advantage of being immediately relevant because the discovered biomarkers are active within the same context of disease encountered in clinical practice. Rather than force our current understanding of the disease mechanisms on the discovery process, we required candidate markers to discriminate risk class (that is, high or low risk of moderate to severe AKI in 12 to 36 hours). Once the best-performing markers had been identified, we tested their performance in a second group of adult critical care patients, thus requiring them to show robust utility across multiple institutions and patient subtypes. Finally, we subjected the new markers to analyses that tested their ability to enhance discrimination over robust clinical models. For these reasons we believe that these markers are the most promising early markers of AKI reported to date.

We chose to assess risk of moderate to severe AKI rather than all AKI because this severity (corresponding to KDIGO stage 2 and 3) has been shown to be associated with a significantly increased incidence of clinically important outcomes such as need for renal replacement therapy, in hospital death, and persistent renal dysfunction [2,3].

**Figure 5:**
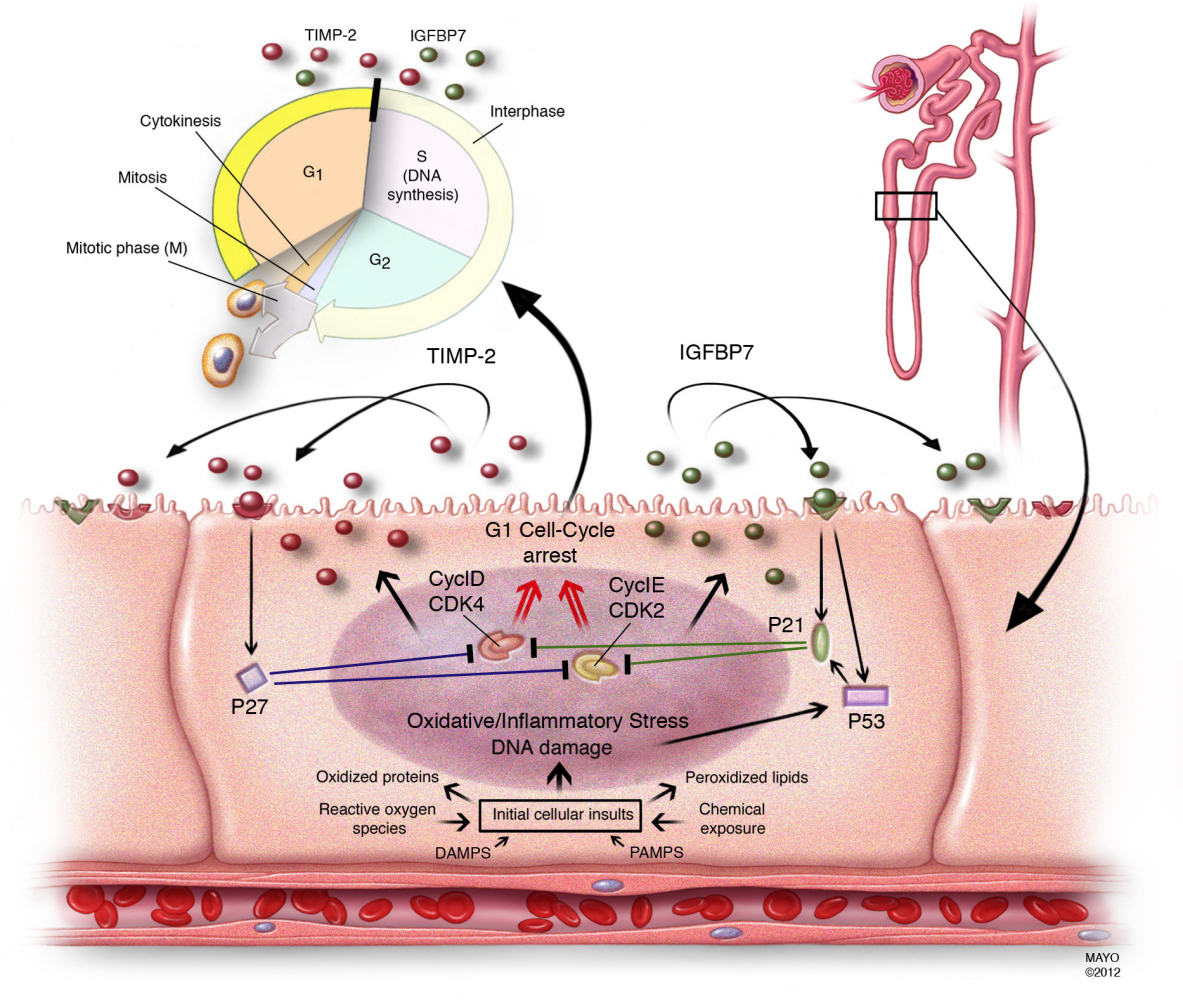
**Proposed mechanistic involvement of the novel biomarkers in AKI: initial tubular cells sustain injury by various insults**. In response to DNA and possibly other forms of damage, IGFBP7 and TIMP-2 are expressed in the tubular cells. IGFBP7 directly increases the expression of p53 and p21 and TIMP-2 stimulates p27 expression. These effects are conducted in an autocrine and paracrine manner via IGFBP7 and TIMP-2 receptors. The p proteins in turn, block the effect of the cyclin-dependent protein kinase complexes (CyclD-CDK4 and CyclE-CDK2) on the cell cycle promotion, thereby resulting in G_1 _cell cycle arrest for short periods of time presumably to avoid cells with possible damage from dividing. AKI, acute kidney injury; IGFBP7, insulin-like growth factor-binding protein 7; TIMP-2, tissue inhibitor of metalloproteinases-2.

Our results also help shed additional light on the pathogenesis of AKI. Our analysis included more than 300 molecules representing multiple biologic pathways believed to be important in the pathogenesis of AKI. It is notable therefore that IGFBP7 and TIMP-2 are both involved with the phenomenon of G_1 _cell cycle arrest during the very early phases of cell injury (Figure 5) [21-24]. AKI engages a series of extremely complex cellular and molecular pathways involving endothelial, epithelial, inflammatory, and interstitial cells. These mechanisms include cell cycle, immunity, inflammation, and apoptosis pathways. Recently, it has been shown that, similar to other epithelia, renal tubular cells enter a short period of G_1 _cell-cycle arrest following injury from experimental sepsis [25] or ischemia [26]. It is believed that this prevents cells from dividing when the DNA may be damaged and arrests the process of cell division until the damage can be repaired lest resulting in the cell's demise or senescence [22]. Interestingly, these markers perform very well in patients with sepsis (AUC 0.82) and post-surgery (AUC 0.85) (Figure S1 in Additional file 1). Also of interest is that IGFBP7 is superior to TIMP-2 in surgical patients while TIMP-2 is best in sepsis-induced AKI (Figure S2 in Additional file 1). These differences may underlie subtle but important mechanistic differences between various etiologies of AKI, and the two biomarkers are involved in slightly different, pathways (Figure 5). These results also support the use of the two biomarkers, which together provide the most consistent result across cohorts.

Markers of cell-cycle arrest such as TIMP-2 and IGFBP7 may signal that the renal epithelium has been stressed and has shut down function but may still be able to recover without permanent injury to the organ. Importantly, both TIMP-2 and IGFBP7 appear to be able to signal in autocrine and paracrine fashions [24,27-30] thus spreading the 'alarm' from the site of injury. In terms of timing, this signal could be ideal as it may be early enough that treatment can still alter the outcome - further study will be required to test this hypothesis. Finally, TIMP-2 and IGFBP7 are known to be involved in the response to a wide variety of insults (inflammation, oxidative stress, ultraviolet radiation, drugs, and toxins) [16,23,24]. This may help explain why they correspond to risk for AKI, a syndrome known for its multiple etiologies even in the same patient.

Our study has important limitations. Although we measured more than 300 candidates in our discovery study, many taken from unbiased 'omics' approaches, our list is by no means exhaustive. Furthermore, because we felt that the most important unanswered question was early risk stratification, we chose to study patients without evidence of AKI and sought to predict its clinical manifestation over the next 12 hours. Thus, we emphasized molecules with a rapid response to injury. We recognize that progression of disease and recovery are also important clinical questions and our results do not directly address these areas.

## Conclusions

Urine TIMP-2, and IGFBP7, two novel biomarkers for risk stratification of AKI, were discovered and validated in more than 1,000 critically ill patients. These markers performed better than any other biomarker reported to date, showed significant enhancement over clinical variables, are mechanistically relevant, and can be easily measured with existing technology. Indeed, we chose to validate the two-marker panel ([TIMP-2]·[IGFBP7]) using a clinical rather than a research assay so as to facilitate rapid translation into clinical practice. The introduction of this new test should significantly improve the ability of physicians caring for critically ill patients to identify risk of impending AKI; and also facilitate future AKI research by permitting more accurate identification of high-risk patients for enrollment into intervention trials.

## Key messages

• Urine insulin-like growth factor-binding protein 7 (IGFBP7) and tissue inhibitor of metalloproteinases-2 (TIMP-2) are new biomarkers for AKI and perform better than existing markers for predicting the development of moderate or severe AKI (KDIGO stage 2 or 3) within 12 hours of sample collection.

• [TIMP-2]·[IGFBP7] significantly improved risk stratification when added to a nine-variable clinical model when analyzed using Cox proportional hazards model, generalized estimating equation, integrated discrimination improvement or net reclassification improvement.

• Risk for major adverse kidney events (death, dialysis or persistent renal dysfunction) within 30 days (MAKE_30_) elevated sharply for [TIMP-2]·[IGFBP7] above 0.3 and doubled when values were >2.0.

• Both IGFBP7 and TIMP-2 are inducers of G_1 _cell-cycle arrest, a key mechanism implicated in AKI.

## Abbreviations

AKI: Acute kidney injury; AUC: area under the receiver-operating characteristics curve; cfNRI: category-free net reclassification improvement; IDI: integrated discrimination improvement; IGFBP7: insulin-like growth factor-binding protein 7; IL-18: interleukin-18; KIM-1: kidney injury molecule-1; L-FABP: liver fatty acid-binding protein; MAKE: major adverse kidney event; NGAL: neutrophil gelatinase-associated lipocalin; pi-GST: pi-Glutathione S-transferase; TIMP-2: tissue inhibitor of metalloproteinases-2.

## Competing interests

KK, AA-K, TA, SB, MB, AB, CC, DD, LF, MG, JH, PH, EH, OJ, PK, JLK, ML, PM, SM, MO, TR, JS, AS, JV, LW, MW, GW and KZ report no conflicts of interest. AA is on the Scientific Advisory Board for Ferrer and for Gambro. RB has received grants for research from Alere and Abbott Diagnostics. LC has consulting agreements with Abbott Medical, Affymax, Alere, AM Pharma, Astute Medical, Covidien, Gambro, Nxstage Medical, Sanofi, and Bonner Kiernan Law Offices. LC has applied for research support from Eli Lilly and owns stock in MAKO Corporation for an orthopedic surgical robot. TF has received grant support from B. Braun Meslungen, Fresenius and Roche. TF has received lecture fees from Alexion, Amgen, B. Braun Meslungen, BMS, Cellpharm, Novartis, Otsuka, Roche, and Teva. KG has received research grants from Spectral Diagnostics. MH has received lecture fees from Abbott Diagnostics and Alere, both involved in the development of NGAL. MJ has received speaker fees from Astute Medical, Baxter, Fresenius, and Gambro. MJ has received consulting fees from Gambro, Baxter, Fresenius, and AM Pharma. DL is a consultant for Astute Medical for which he has received compensation in the form of stock options. GM has received research grants and payment for lectures from B. Braun Meslungen and from CytoSorbents. NS has received research funding from Alere, Cheetah Medical, and Thermo Fisher. AS has received fees for expert testimony from Abbott Laboratories and is a Medical Advisory Board member for FAST diagnostics for optical GFR measurement and a Scientific Advisory Board member for NxStage Medical for CRRT in the ICU. CV has received research funds from Gambro. JK has received consulting fees from Astute Medical, Alere, Opsona, Aethlon, AM Pharma, Cytosorbents, venBio, Gambro, Baxter, Abbott Diagnostics, Roche, Spectral Diagnostics, Sangart, and Siemens. JK has also received research grants from Astute Medical, Alere, Cytosorbents, Gambro, Baxter, Kaneka, and Spectral Diagnostics, and has licensed unrelated technologies through the University of Pittsburgh to Astute Medical, Cytosorbents and Spectral Diagnostics.

## Authors' contributions

MW and JS had full access to all of the data in the study and take responsibility for the integrity of the data and the accuracy of the data analysis. *Design and conduct of the study: *JK, LC and KK designed the study in conjunction with the sponsor. KK was the Principal Investigator for the Discovery study and JK and LC were the Principal Investigator and Co-Principal Investigator, respectively, for the Sapphire study. *Data collection: *KK, DL, and LW enrolled subjects, gathered and interpreted the data for the discovery study. AA-K, TA, AA, SB, MB, AB, RB, CC, DD, TF, LF, MG, KG, MH, PH, EH, OJ, MJ, PK, JLK, KK, ML, GM, PM, SM, MO, TR, AS, NS, AS, JV, CV, GW, and KZ enrolled subjects, gathered and interpreted the data for the Sapphire study. *Management: *Sapphire study clinical data were managed by a contract research organization (Synteract, Carlsbad, CA, USA) and could be entered or modified only from the collection site with the site investigator's permission. Discovery study clinical data were managed by Astute Medical (San Diego, CA, USA). *Statistical analysis: *MW, JS, and JH performed the statistical analysis for the Sapphire study. Astute Medical performed the statistical analysis for the Discovery study. *Interpretation of the data: *All authors reviewed the data and participated in discussions related to interpretation. *Preparation, review or approval of the manuscript: *JK, KK, LC, AS, and MW wrote the paper. All authors reviewed and edited the paper and have seen and approved the final draft.

## Supplementary Material

Additional file 1 **Supplemental data**. Supplemental and supporting data unrelated to the primary analyses are provided in Additional File 1. The additional data file also contains lists of study personnel, detailed laboratory methods and sensitivity analyses. Click here for file
