# Supplementary material for: Discovery and validation of cell cycle arrest biomarkers in human acute kidney injury
Source: Crit Care. 2013 Feb 6;17(1):R25. doi: 10.1186/cc12503 (PMC4057242; doi:10.1186/cc12503)
Supplement: Additional file 1 — Supplemental data. Supplemental and supporting data unrelated to the primary analyses are provided in Additional File 1. The additional data file also contains lists of study personnel, detailed laboratory methods and sensitivity analyses. [file cc12503-S1.DOC]

**Supplementary Appendix**

**Discovery and validation of cell cycle arrest biomarkers in human acute kidney injury**

Kianoush B Kashani MD, Ali Al-Khafaji MD, Thomas Ardiles MD, Antonio Artigas MD, Sean M Bagshaw MD, MSc, Max Bell MD, PhD, Azra Bihorac MD, Robert Birkhahn MD, Cynthia M Cely MD, Lakhmir S Chawla MD, Danielle L Davison MD, Thorsten Feldkamp MD, Lui Forni MD, Michelle Ng Gong MD, MS, Kyle Gunnerson MD, Michael Haase MD, James Hackett PhD, Patrick M Honore MD, PhD, Eric AJ Hoste MD, PhD, Olivier Joannes-Boyau MD, Michael Joannidis MD, Patrick Kim MD, Jay L Koyner MD, Daniel T Laskowitz MD, Matthew Lissauer MD, Gernot Marx MD, Peter A McCullough MD, PhD, MPH, Scott Mullaney MD, Marlies Ostermann MD, Thomas Rimmelé MD, Nathan I Shapiro MD, Andrew D Shaw MD, Jing Shi PhD, Amy M Sprague MD, Jean-Louis Vincent MD, Christophe Vinsonneau MD, Ludwig Wagner MD, Michael G Walker PhD, R Gentry Wilkerson MD, Kai Zacharowski MD, PhD, John A Kellum MD.

Contents

[**Sapphire and Discovery Study Sites and Personnel 2**](#__RefHeading___Toc345667399)

[**Discovery Studies 4**](#__RefHeading___Toc345667400)

[**Sapphire Study 5**](#__RefHeading___Toc345667401)

[**Blood and Urine Samples (Sapphire study*)* 6**](#__RefHeading___Toc345667402)

[**Clinical Endpoints and Sensitivity Analyses 7**](#__RefHeading___Toc345667403)

[**Laboratory Methods 9**](#__RefHeading___Toc345667404)

[**Statistical Analysis 9**](#__RefHeading___Toc345667405)

[**Biomarker Discovery and Selection Rationale 10**](#__RefHeading___Toc345667406)

[**Urinary [TIMP-2]*•*[IGFBP7] Values In Apparently Healthy Donors 11**](#__RefHeading___Toc345667407)

[**Tables and Figures 12**](#__RefHeading___Toc345667408)

[**Supplemental References 24**](#__RefHeading___Toc345667409)

**Sapphire and Discovery Study Sites and Personnel**

Sapphire Principal Investigator: John A Kellum, MD

Sapphire Co-Principal Investigator: Lakhmir S Chawla, MD

Discovery Study Principal Investigator: Kianoush B Kashani, MD

Sapphire Study Enrolling Sites:

Arizona: *Maricopa Integrated Health System* – Thomas Ardiles, MD; Tera Williamson, BS.

California: *University of California San Diego* – Scott Mullaney, MD.

District of Columbia: *George Washington University Medical Center* – Danielle L Davison, MD; Christina Seneff; Ermira Brasha-Mitchell.

Florida: *Bruce W. Carter Department of Veterans Affairs Medical Center* – Cynthia Cely, MD; Roland M Schein, MD; Andrew Quartin, MD, MPH. *Tampa General Hospital* – R Gentry Wilkerson, MD; James Wilson, MD; Christina Targal, EMT-B. *University of Florida* – Azra Bihorac, MD; Minal Patel, MD.

Georgia: *Joseph M Still Research Foundation* – Amy M Sprague, MD.

Illinois: *University of Chicago* – Jay L Koyner, MD; Sharon Trevino, RN.

Maryland: *University of Maryland School of Medicine* – Matthew Lissauer, MD; Jessica Warren; Holly Howes.

Massachusetts: *Beth Israel Deaconnes Medical Center* – Nathan I Shapiro, MD.

Michigan: *St John Providence Health System* – Peter A McCullough, MD, MPH.

Minnesota: *Mayo Clinic* – Kianoush B Kashani, MD; Laura Hanson.

New York: *Montefiore Medical Center* – Michelle Ng Gong, MD; Mirian Martinez, RN; John Salcedo. *New York Methodist Hospital* – Robert Birkhahn, MD; Paris Datillo, RN.

North Carolina: *Durham VAMC* – Andrew D Shaw, MD; Becky Perfect; Lizzie Rogers.

Pennsylvania: *Hospital of the University of Pennsylvania* – Patrick Kim, MD. Babak Sarani, MD; Joy Steele, RN. *University of Pittsburgh* – Ali Al-Khafaji, MD; Denise Scholl, CRC.

Virginia: *Virginia Commonwealth University Medical Center* – Kyle Gunnerson, MD; Tamara Ponton, RN; Jennifer Chadbourne.

Austria: *University Clinic for Internal Medicine* – Michael Joannidis, MD; Georg Lehner, MD.

Belgium: *Erasme University Hospital* – Jean-Louis Vincent, MD. *Ghent University Hospital* – Eric AJ Hoste, MD. *UZB-VUB University Hospital* – Patrick M Honore, MD, PhD; Herbert D Spapen, MD, PhD; Rita Jacobs, MD; Jouke De Regt, MD; Elisabeth De Waele, MD; Marie-Claire Van Malderen, RN; Godelieve Opdenacker, RN; Marijke de Mars, RN.

Canada: *University of Alberta* – Sean M Bagshaw, MD.

France: *Edouard Herriot Hospital* – Thomas Rimmelé, MD; Rita Mendes Oliveira; Catherine Jouvène. *Hospital Marc Jacquet* – Christophe Vinsonneau, MD; Lormail Cecile. *University Hospital of Bordeaux* – Olivier Joannes-Boyau, MD; A Dewitte, MD; J Coquin, MD.

Germany: *Otto-von-Guericke-Universitat Magdeburg* – Michael Haase, MD; Julia Horlbeck; Caroline Tiedemann. *Universitätsklinikum der RWTH Aachen* – Gernot Marx, MD; Tobias Schuerholz. *University Hospital Essen, University Duisburg-Essen* – Thorsten Feldkamp, MD; Michael Volesk; Bartosz Tyczynski. *University Hospital Frankfurt* – Kai Zacharowski, MD; Tobias Bingold, MD.

Spain: *Sabadell Hospital* – Antonio Artigas, MD; Ignacio Martin-Loeches, MD; Gisela Gillis, RN.

Sweden: *Karolinska University Hospital* – Max Bell, MD, PhD; Daniella Johansson, MSc; Claire Rimes-Stigare, MD.

UK: *Guy’s and St Thomas Hospital* – Marlies Ostermann, MD. *Worthing Hospital* – Lui Forni, MD; Jordi Margalef, BSc.

Discovery Study Enrolling Sites:

Minnesota: *Mayo Clinic* – Kianoush B Kashani, MD; Laura Hanson.

North Carolina: *Duke University Medical Center* – Daniel T Laskowitz, MD; Bradley Kolls, MD; Stephanie Macy.

Austria: *Medical University of Vienna* – Ludwig Wagner, MD; Aysegul Ilhan, MD; Christian Zauner, MD.

**Discovery Studies**

Subjects were enrolled at three independent clinical sites in the Discovery studies: Medical University of Vienna (Vienna, Austria), Duke University Medical Center (Durham, NC) and Mayo Clinic (Rochester, MN). Data from the three sites were pooled in the Discovery analyses (see Figure 1 in the manuscript). Baseline characteristics and outcomes are shown in Table S1. Entry criteria were as follows:

Vienna Cohort

### Inclusion Criteria

1. Males and females 18 years of age or older;
2. Expected to be hospitalized in the ICU with sepsis for at least 48 hours after enrollment;
3. Able and willing to provide informed consent for study participation and to comply with all study procedures.

### Exclusion Criteria

1. Already receiving dialysis (either acute or chronic) or in imminent need of dialysis at enrollment;
2. Participation in an interventional clinical study with an experimental therapy within the previous 30 days.
3. Known infection with human immunodeficiency virus (HIV) or a hepatitis virus.

Duke Cohort

### Inclusion Criteria

a. Males and females 18 years of age or older;

1. Expected to be hospitalized through an acute care setting (ED, Neuroscience ICU, Medical ICU, and Coronary Care Unit) with at least one of the following:

- Risk factor for renal injury (e.g., sepsis, hypotension/shock, major trauma, hemorrhage, scheduled contrast procedure, major surgery);
- Expected to be hospitalized in the ICU for at least 24 hours after enrollment.

*Note: all the enrolled patients were in the ICU*

1. Subject/Legally Authorized Representative able and willing to provide written informed consent for study participation and to comply with all study procedures.

### Exclusion Criteria

1. Institutionalized individuals;
2. Previous renal transplantation;
3. Known acutely worsening renal function prior to enrollment (e.g., documented increase in serum creatinine of at least 25% over the preceding 48 hours);
4. Received dialysis (either acute or chronic) within five days prior to enrollment or in imminent need of dialysis at the time of enrollment;
5. Known infection with human immunodeficiency virus (HIV) or a hepatitis virus.

Mayo Cohort

### Inclusion Criteria

a. Males and females 18 years of age or older;

1. Has at least one of the following acute medical conditions associated with a risk for subsequent AKI:
   - Shock (SBP < 90 mmHg and/or need for vasopressor support to maintain MAP > 60 mmHg and/or documented drop in SBP of at least 40 mmHg);
   - Sepsis;
   - IV Antibiotics ordered in the Computerized Physician Order Entry System within 24 hours of enrollment;
   - Contrast media exposure within 24 hours of enrollment;
   - Increased Intra-Abdominal Pressure (> 8 mm Hg) with acute decompensated heart failure;
   - Severe Trauma as the primary reason for ICU admission and likely to be hospitalized in the ICU for 48 hours after enrollment.
2. Able and willing to provide written informed consent for study participation and to comply with all study procedures.

### Exclusion Criteria

1. Known pregnancy;
2. Institutionalized individuals;
3. Previous renal transplantation;
4. Known acutely worsening renal function prior to enrollment (e.g., any category of RIFLE criteria);
5. Already receiving dialysis (either acute or chronic) or in imminent need of dialysis at the time of enrollment;
6. Known infection with human immunodeficiency virus (HIV) or a hepatitis virus;
7. Meets only the SBP < 90 mmHg inclusion criterion and does not have shock in the attending physician’s or principal investigator’s opinion (i.e., individuals with constitutionally low SBP).

**Sapphire Study**

Subjects in the Sapphire study were enrolled at 35 clinical sites in North America and Europe (see Figure 1 in the manuscript). Exposures and susceptibilities for AKI are shown in Table S2. Receiver-operating characteristics curves and AUCs are shown in Figures S1 and S2, respectively. Cox proportional hazards models of [TIMP-2]*•*[IGFBP7] and serum creatinine are shown in Table S3. Cox proportional hazards models of [TIMP-2]*•*[IGFBP7], serum creatinine and additional clinical covariates are shown in Table S4. Generalized estimating equation (GEE) models of [TIMP-2]*•*[IGFBP7], serum creatinine and additional clinical covariates are shown in Table S5. Integrated discrimination improvement (IDI) and category-free net reclassification improvement (cfNRI) analyses are shown in Table S6 and risk assessment plots are shown in Figure S3. Entry criteria were as follows:

### Inclusion Criteria

1. Males and females 21 years of age or older;
2. Subjects enrolled (first sample collection) from ED or Floor must be admitted to the ICU within 24 hours of enrollment;
3. Subjects enrolled in the ICU must have been admitted to the ICU or transferred into the study ICU from another ICU no more than 24 hours prior to enrollment;
4. Expected to remain in the ICU for at least 48 hours after enrollment;
5. Use of indwelling urinary catheter as standard care expected for at least 48 hours after enrollment;
6. At least one of the following acute conditions within 24 hours prior to enrollment:
   - Respiratory sepsis-related organ failure assessment (SOFA) score of ≥ 2 (PaO_2_/FiO_2_ <300);
   - Cardiovascular SOFA score of ≥ 1 (MAP < 70 mm Hg and/or any vasopressor required).
7. Patient (or authorized representative) able and willing to provide written informed consent for study participation.

### Exclusion Criteria

1. Special populations including women with known pregnancy, prisoners, or institutionalized individuals;
2. Previous renal transplantation;
3. Known moderate to severe AKI prior to enrollment (e.g., Kidney Disease Improving Global Outcomes (KDIGO) stage 2-3);

*Note: original protocol excluded any AKI; the protocol was amended during the study after 222 patients had been enrolled to exclude only KDIGO stage 2 or 3*

1. Already receiving dialysis (either acute or chronic) or in imminent need of dialysis at the time of enrollment;
2. Known infection with human immunodeficiency virus (HIV) or active hepatitis (acute or chronic);
3. Patient meets any of the following:

- Active bleeding with an anticipated need for > 4 units PRBC in a day;
- Hemoglobin < 7 g/dL;
  - Any other condition that in the physician’s opinion would contraindicate drawing serial blood samples for clinical study purposes.

**Blood and Urine Samples (Sapphire study*)***

### Study-specific blood samples were obtained via direct venipuncture, via other available venous access (e.g., an existing femoral sheath, central venous line, peripheral intravenous line, or hep-lock) or via an indwelling arterial line. Blood was collected in EDTA-anti-coagulated whole blood tubes (for plasma) and in clot activator blood collection tubes (for serum). Plasma and serum were prepared by standard methods (centrifugation for 15 minutes at 1,600 x g and for 10 minutes at a minimum of 1,300 x g after clotting, for plasma and serum, respectively) and aliquots were frozen (on dry ice or liquid nitrogen) within about an hour of collection (median and interquartile range for time to freezing of plasma samples were 0.8 hours and 0.5-1.0 hours, respectively). Samples were shipped on dry ice and stored at ≤ -70⁰ C. Samples were thawed immediately prior to measurement. A sample was considered unevaluable if it contained insufficient volume (blood collection tube contained less than half the fill volume), was severely hemolyzed, or was not properly frozen upon arrival after shipping.

Study-specific urine samples were collected in standard (non-coated) specimen cups. For patients with an in-dwelling bladder catheter, urine could be collected directly from the catheter or from the collection bag provided the bag was emptied first and a fresh sample collected. For patients in which the in-dwelling catheter had been removed, urine was collected by the clean catch method. Samples were centrifuged (10 minutes at 1,000x g) to remove any cells or other debris, and aliquots were frozen (on dry ice or liquid nitrogen) within about an hour of collection (median and interquartile range for time to freezing of urine samples were 0**.**8 hours and 0**.**6-1**.**0 hours, respectively). Samples were shipped on dry ice and stored at ≤ -70⁰ C. Samples were thawed immediately prior to measurement. A sample was considered unevaluable if not properly frozen upon arrival after shipping.

**Clinical Endpoints and Sensitivity Analyses**

AKI status in the Discovery study was classified using the original RIFLE criteria [1]. AKI status in the Sapphire study was classified using the recently harmonized RIFLE/AKIN criteria, as summarized in Recommendations 2**.**1**.**1 and 2**.**1**.**2 of the KDIGO consensus guidelines [2]. AKI stage was the highest determined from serum creatinine (sCr) or hourly urine output (UO), using sCr and UO values as available from the hospital record. All sCr values (including inpatient and outpatient values) available in the hospital record for six months prior to enrollment through study day 30 (or hospital discharge or death if earlier) were collected. All hourly urine output values available in the hospital record from up to 24h prior to enrollment through study day seven were collected. To be considered evaluable, a patient must have had at least two sCr values (including one prior to enrollment) or six hours of UO data available. All data used in the calculation of RIFLE/AKIN levels were 100% source verified from the hospital record by a clinical monitor.

RIFLE/AKIN criteria require a reference sCr value to diagnose and stage AKI. Various methods for determining the reference sCr values have been described and there is no method that is universally accepted [2-4]. The primary method used herein (Method A) was defined during the Discovery study, and was therefore prospectively defined and locked prior to the start of the Sapphire analysis. We performed sensitivity analysis (Table S7) in which the reference sCr values were calculated several ways incorporating methods recently described [3,4].

The sCr reference value in Method A (primary analysis) was calculated as follows:

1. Median of all values available in the time range starting six months and ending six days prior to enrollment, IF at least five values were available, OR IF the number of values available exceed the number available in the time range starting five days prior to enrollment and ending at enrollment; ELSE,
2. Nadir value in the time range starting five days prior to enrollment and ending at enrollment, IF at least one value was available; ELSE,
3. Enrollment value (defined as the value obtained closest to the time of enrollment).

A minimum of five values and the median rather than mean were specified for step (1) to minimize the possible impact of any isolated (single point) acute elevations of sCr that may have occurred in the six months prior to enrollment (since the values available included inpatient values). For step (2), the nadir (rather than median) value was specified to minimize the possible impact of any acute elevations in sCr during the five days prior to enrollment.

The sCr reference value in Method B was calculated as follows:

1. Most recent value from at least seven days prior to hospital admission, IF at least one value was available; ELSE
2. Nadir value from seven days prior to hospital admission to enrollment, IF at least one value was available; ELSE
3. Value closest to enrollment

The sCr reference value in Method C was calculated as follows:

1. Most recent value from at least seven days prior to hospital admission, IF at least one value was available; ELSE
2. Patient excluded from the analysis (i.e., if no pre-hospital baseline could be calculated)

In methods B and C, the time of at least seven days prior to hospital admission (step 1) to define a pre-admission baseline period was chosen to be consistent with a recently described method [4]. Siew et al [4] found that the mean of outpatient sCr values from at least seven days prior to hospital admission yielded the best agreement (intraclass correlation coefficient, ICC = 0**.**91; 95% CI = 0**.**88-0**.**92) with an adjudicated reference standard, but that the most recent inpatient or outpatient value from the same period agreed almost as well (ICC = 0**.**88; 95% CI = 0**.**85-0.91) and was within the confidence interval of the method using the mean. We chose the latter (most recent value) to be consistent with Siew et al [4] since the sCr recorded in the Sapphire study included both inpatient and outpatient values. The use of a nadir in-hospital value when a pre-hospital baseline is not available has been described in several publications [3,5]. We chose to use a nadir in-hospital value (rather than median or mean) if a pre-hospital baseline value was not available (step 2 of methods A and B) to mitigate for the possibility that patients could have had an elevation of sCr associated with early or mild AKI prior to enrollment (i.e., up to KDIGO stage 1, which was not excluded). Siew et al [3] found that use of a nadir in-hospital sCr value led to about 15% of patients misclassified as positive for any AKI and about 4**.**6% of patients misclassified as negative for any AKI as compared with using a known outpatient sCr baseline value. However, they found the misclassification for KDIGO stage 2 or 3 (the primary Sapphire endpoint) to be modest with only about 2**.**8% misclassified as positive and 1**.**5% misclassified as negative; a result also seen in a prior study by Zavada et al [6]. Furthermore, Siew et al [3] did not incorporate UO in their determination of AKI stage, and the incorporation of UO will tend to mitigate inaccuracies associated with the sCr reference value [6].

RIFLE/AKIN criteria require at least 12 consecutive hours of oliguria (< 0**.**5 mL/kg per hour) to define AKI of at least stage 2. Missing (from the hospital record) hourly UO values were handled as follows: (1) if urine volume for a time interval without recorded hourly UO was included in a subsequent cumulative total, the cumulative total divided by the number of hours in the time interval was assigned to each hour; (2) if urine volume for a time interval without recorded hourly UO was not included in a subsequent cumulative total, or if the urinary catheter was removed, the patient was considered to be non-oliguric in the time interval. Anuria was defined to be less than 50 mL of UO in a 12 hour period, if UO data were available for the entire period.

In the Sapphire study analysis, each biomarker sample was classified as positive or negative for the endpoint of AKI (KDIGO stage 2 or 3) for the 12 hour period following the collection of the sample. We selected a period of 12 hours because critically ill patients are in a dynamic health state with rapidly changing acute risk factors (exposures), kidney status and biomarker levels, and 12 hours is a relevant time for assessment and intervention. A sample was classified as positive for the endpoint if any of the following were met during the 12 hours: (1) sCr at least 100% above the reference value; (2) at least 12 consecutive hours of oliguria (UO <0**.**5 mL/kg per hour) for any 12 hour period ending within the prediction window; (3) anuria for any 12 hour period ending within the prediction window; (4) 4 mg/dL absolute sCr value within the prediction window and at least a 0**.**3 mg/dL increase in sCr in the previous 48 hours or at least a 50% increase in sCr above the reference value; (5) any renal replacement therapy on the day of sample collection. Serum creatinine values were linearly interpolated between the measured values available in the hospital record. Samples were classified as negative for the endpoint if none of the conditions (1)-(5) were met during the 12 hours following collection of the sample, even if a previous sample from the same patient had been classified as positive. Nominal sample collection times were at enrollment and 12 hours after enrollment, with a variation of about ± 6h for the second sample collection. Therefore, all samples collected within 18h of enrollment were included in the bootstrap analyses.

Patients are often admitted to the ICU without a reference sCr value and with no or incomplete UO data, and diagnosis and classification by AKI stage for the first day in the ICU must be refined over time (often days) as more data become available [2]. Therefore, patients could be properly enrolled in the Sapphire study but later determined to have already had KDIGO stage 2 or 3 at the time of enrollment and/or sample collection, unbeknownst to the clinical team. We therefore included all properly collected samples in the bootstrap analyses, even if retrospective RIFLE/AKIN analysis showed the patient was already at the endpoint prior to sample collection, since this corresponds to the situation likely to prevail in clinical practice. We performed sensitivity analyses (Table S7) in which patients already at the endpoint prior to enrollment or sample collection were excluded, and in which all but the first sample (i.e., the baseline enrollment sample) were excluded. These exclusion analyses showed no substantial impact on the results.

**Laboratory Methods**

Biomarkers were measured with single or multiplexed immunoassays using standard ELISA, Luminex 200 (Luminex, Austin TX), MSD SECTOR Imager 6000 (Meso Scale Discovery, Gaithersburg, MD) or Astute140™ Meter (Astute Medical, San Diego CA, not available in the US at time of manuscript submission) platforms. Immunoassays were either developed by Astute Medical or obtained from vendors and used as recommended by the vendor or modified to optimize performance. Modifications included addition of an agent (Heterophilic Blocking Reagent-1, P/N 3KC533, Scantibodies Laboratory Inc, Santee, CA) to plasma samples to block heterophilic antibodies and modification of incubation times or wash buffers. Novel biomarkers were measured with research assays (TIMP-2: R&D Systems P/N LKT003, Minneapolis, MN; IGFBP7: Millipore P/N HIGFBP-53K, Billerica, MA) in the Discovery study and with the NephroCheck® Test (Astute Medical, San Diego, CA) in the Sapphire study. The NephroCheck Test was developed to simultaneously measure the two top performing biomarkers from the Discovery study using a platform that can be used clinically. Previously described biomarkers of AKI were measured with commercially available assays (NGAL: BioPorto Diagnostics Cat# 036, Gentofte, Denmark; Cystatin C: Millipore P/N HKTX2MAG-38K, Billerica, MA; KIM-1 and IL-18: Meso Scale Discovery P/N N05ZA-1, Gaithersburg, MD; Pi-GST: Argutus Medical Cat# BIO85, Dublin, Ireland; L-FABP: CMIC Co., Cat# Z-001, Tokyo, Japan). Serum creatinine for the covariate analysis of [TIMP-2]*•*[IGFBP7] (Cox proportional hazards and generalized estimating equation models and integrated discrimination improvement analysis) was measured at a central lab (LabCorp, San Diego, CA) in serum samples that were collected at the same times as the urine samples analyzed by the NephroCheck Test (paired serum and urine samples).

# Statistical Analysis

Statistical analyses were performed using SAS 9**.**3 and R 2**.**12. For all analyses, two-sided P values less than 0**.**05 were considered statistically significant. Categorical variables were analyzed using Fisher Exact test or logistic regression. Area under the receiver operating characteristic curve (AUC) was calculated as empirical AUC with bootstrap confidence intervals to handle subjects with more than one sample collected within 18 hours of enrollment [7]. Differences between AUC's were tested using bootstrap sampling. Time to event analyses used Cox Proportional Hazards regression. Tests of trend in relative risk across tertiles used the Jonckheere Terpstra [8].

For the Sapphire study, Cox Proportional Hazard (PH) regression (Tables S3 and S4) was used to evaluate the relationship between biomarker measurements and the time to the first KDIGO stage 2 or 3 event within 12 hours of biomarker collection, where biomarker measurements (paired measurements of [TIMP-2]*•*[IGFBP7] and serum creatinine) were included as time dependent covariates and the censoring time after each biomarker collection was the time at which the earliest of the following occurred: the last sCr or UO collection, the next biomarker collection, or 12 hours after the current biomarker collection. Other parameters were included as fixed covariates. The adequacy of fit and the proportional hazards assumption of the model were evaluated separately for each biomarker using cumulative Martingale residuals [9]. Biomarkers not well fit or violating the proportional hazards assumption on the original scale were first transformed with the log. If log transformed measurements did not yield both adequate fit and proportional hazards, a series of restricted cubic spline transforms [10,11], each utilizing an additional degree of freedom, were sequentially investigated until an acceptable transformation was identified. The statistical significance of each time dependent covariate was assessed using the likelihood ratio chi-square test.

The Cox proportional hazards methodology was used to examine the enhancement of clinical models by the addition of [TIMP-2]*•*[IGFBP7]. First, we analyzed serum creatinine and [TIMP-2]*•*[IGFBP7] (Table S3), since serum creatinine is a ubiquitous lab test ordered at least daily as standard care in the ICU. Next, we included additional clinical covariates in the clinical model (Table S4). All covariates shown in Table 1 of the main manuscript and Table S2 with p-value < 0.1 were included, as was chronic kidney disease.

We also performed Integrated Discrimination Improvement (IDI) and category-free (continuous) Net Reclassification Improvement (cfNRI) analyses (Table S6) [12] to investigate the enhancement of a clinical model (Table S5) by the addition of [TIMP-2]*•*[IGFBP7]. Similar analyses have been reported previously for several existing AKI biomarkers [13]. Confidence intervals for IDI and cfNRI were estimated using bias-corrected accelerated (BCa) bootstrap [14]. The reference model (Table S5) for IDI and cfNRI included all covariates shown in Table 1 of the main manuscript and Table S2 with p-value < 0.1 as well as chronic kidney disease. The new model for IDI and cfNRI (Table S5) included the covariates in the reference model with the addition of [TIMP-2]*•*[IGFBP7]. Risk estimates for both the reference and new model were calculated using Generalized Estimating Equations (GEE) implemented in the geepack R package [15].

Enhancement of the reference clinical model (Table S5) by the addition of [TIMP-2]*•*[IGFBP7] was also examined graphically with Risk Assessment plots (Figure S3) [16].

Sample size calculations for the Sapphire study were based on AUC estimates and projected AKI rates from the Discovery study with the objective of obtaining a narrow confidence interval around the AUC estimate for the primary endpoint. Additionally, a large number of subjects with AKI was desired to increase the likelihood of capturing a representative range of AKI etiologies. Biomarker testing was stopped when approximately 100 subjects that reached the primary endpoint were obtained, yielding a half-width of the 95% CI < 0.04 for the AUC of [TIMP-2]*•*[IGFBP7].

# Biomarker Discovery and Selection Rationale

340 candidate biomarkers were identified for analysis in the Discovery study through several approaches, including developing hypotheses based on the pathophysiology of AKI and licensing proprietary targets that had been identified by proteomic and genomic tools in models of AKI. The group of candidates broadly covered AKI pathophysiology, including inflammation, apoptosis, necrosis, endothelial injury, cell-cell and cell-matrix adhesion, cyto-protection, oxidative processes, and cell cycle modulation. Proteins expressed in the kidney and peripherally (e.g., in leukocytes) were included in the analyses.

The 340 candidate biomarkers were analyzed by immunoassay in the Discovery cohorts. Data from all three Discovery cohorts (Vienna, Duke, and Mayo, see Figure 1 in the manuscript) were pooled in this analysis. We chose to pool data from several cohorts in order to enhance the diversity of subjects in the analysis, so that robust biomarkers were selected that work in a complex, heterogeneous patient population. AKI was classified by the original RIFLE criteria using serum creatinine and urine output values available in the hospital record. Patients were dichotomized according to whether they did or did not have AKI of at least RIFLE stage I within seven days of enrollment. For patients who reached this RIFLE I or F endpoint, urine and plasma samples collected between 12 and 36 hours prior to the first occurrence of the endpoint were analyzed. Hence, the analysis was focused on detecting moderate or severe AKI between 12 to 36 hours prior to clinical manifestation. For patients who did not reach this endpoint, multiple samples collected within seven days of enrollment were analyzed. Area under the receiver-operating characteristics curve (AUC) for patients that reached the endpoint vs. those that did not reach the endpoint was estimated for each candidate biomarker. Biomarkers were ranked by p-value for AUC ≠ 0**.**5. Because the biomarkers were to be validated in a second independent study, corrections based on estimations of the false discovery rate were not applied.

Urinary biomarkers with the least significant univariate p-values were eliminated in four phases. In the first phase, all 340 candidate biomarkers were analyzed in samples from at least 93 patients. In the second, third, and fourth phases, 257, 151, and 109 candidate biomarkers, respectively, were analyzed in additional samples, corresponding to cumulative totals of ≥ 200, ≥ 367, and ≥ 487 patients, respectively. We followed a similar process for candidates analyzed in plasma, except only two phases were completed before plasma was eliminated from consideration because of the superiority of the novel urinary biomarkers. The exception is that previously described plasma biomarkers (e.g. pNGAL) were measured for the entire Discovery cohort for purposes of comparison with the best novel urinary biomarkers.

In addition to ranking biomarkers by univariate AUC, combinations of biomarkers were explored. A conservative and robust algorithm for combining biomarkers (multiplication of biomarker concentrations) without any derived parameters to fit was employed in a forward selection methodology to ameliorate the risk of significant over-training and over-estimation of performance. At each phase of biomarker elimination, all possible combinations of two-four biomarkers (novel or previously described) were ranked to ensure that any biomarker that might contribute in top-performing combinations of biomarkers was retained. Combining more than two-three biomarkers together typically did not result in substantially higher AUC.

In order to maximize the probability that our results would validate in independent cohorts of patients, we selected the biomarkers with the highest univariate AUCs and carefully considered the number of biomarkers to validate in combination. Combinations of three biomarkers were considered but rejected for insufficient increase in AUC compared with combinations of two biomarkers. IGFBP7 and TIMP-2 were ranked first and second, respectively, by point estimate of AUC and p-value. Results for TIMP-2, IGFBP7, their combination, and existing biomarkers measured in the Discovery cohorts are shown in Table S8. The table shows the AUCs for the endpoint used to rank biomarkers, RIFLE I or F clinically manifesting 12-36 hours after collection of the samples. The table also shows the AUCs for the endpoint of RIFLE I or F within 12 hours after sample collection (for samples collected within 18 hours of enrollment). The latter time period is a practical and clinically relevant time for assessment of critically ill patients, who typically have rapidly changing health status and require rapid assessment of AKI risk when they enter the ICU. The results for the two different analyses illustrate why the combination (multiplication of the concentrations) of the two novel biomarkers was selected for development and validation of the NephroCheck Test.

# Urinary [TIMP-2]*•*[IGFBP7] Values In Apparently Healthy Donors

Apparently healthy subjects were enrolled at six centers (central recruiting vendors for clinical trials). Inclusion criteria were: (a) Apparently healthy adults (age ≥ 21 years) and (b) Provide written informed consent for study participation. Exclusion criteria were: (a) Any known or suspected acute illness or condition - including acute infections – at the time of enrollment or within the previous 30 days; (b) Any known or suspected significant chronic medical conditions (such as diabetes, coronary artery disease, renal insufficiency, hypertension, hypercholesterolemia, chronic inflammatory diseases [e.g., rheumatoid arthritis], cancer, etc); (c) Trauma-related surgery within the last 6 months; (d) Any surgery, hospitalization or institutionalization (such as in a nursing home) during the previous 3 months; (e) Received any blood product transfusion within the previous 2 months; (f) Pregnant women or children; (g) Prisoners or institutionalized individuals and (h) Already provided blood or urine samples for this study. Urine specimens collected from a cohort of 383 subjects were analyzed for [TIMP-2]*•*[IGFBP7]. For this cohort, 45.6% were male, 68.1% were white/Caucasian and the mean age (± SD) was 57 (± 16) years. Box plots showing urine [TIMP-2]*•*[IGFBP7] values for the apparently healthy donors and Sapphire patients for comparison are shown in Figure S4. The distribution of [TIMP-2]*•*[IGFBP7] values for apparently healthy donors and critically ill Sapphire patients without AKI are almost identical. This result shows that [TIMP-2]*•*[IGFBP7] is unaffected (not elevated) by the various non-AKI critical illnesses, conditions and comorbidities present in the Sapphire population.

**Tables and Figures**

Table S1. Baseline Characteristics and Outcomes for Discovery Study Cohorts.

|  | **Cohort** | | |  |
| --- | --- | --- | --- | --- |
|  | **Vienna** | **Duke** | **Mayo** | **All** |
| **Number of Patients** | 134 | 123 | 265 | 522 |
| **Gender** |  |  |  |  |
| **Male N (%)** | 86 (64%) | 65 (53%) | 151 (57%) | 302 (58%) |
| **Female** | 48 (36%) | 58 (47%) | 114 (43%) | 220 (42%) |
| **Age** |  | | | |
| **median yrs (IQR)** | 61 (50 - 70) | 60 (50 - 71) | 66 (56 - 76) | 64 (53 - 73) |
| **Race** |  | | | |
| **White N (%)** | 132 (99%) | 95 (77%) | 248 (94%) | 475 (91%) |
| **Black** | 0 (%) | 28 (23%) | 3 (1%) | 31 (6%) |
| **Other/Unknown** | 2 (1%) | 0 (%) | 14 (5%) | 16 (3%) |
| **Reason for Hospital Admission^1^** |  | | | |
| **Respiratory N (%)** | 33 (25%) | 53 (43%) | 59 (22%) | 145 (28%) |
| **Surgery** | 9 (7%) | 15 (12%) | 111 (42%) | 135 (26%) |
| **Cardiovascular** | 22 (16%) | 43 (35%) | 54 (20%) | 119 (23%) |
| **Sepsis** | 70 (52%) | 12 (10%) | 26 (10%) | 108 (21%) |
| **Neurological** | 9 (7%) | 34 (28%) | 8 (3%) | 51 (10%) |
| **Trauma** | 0 (%) | 6 (5%) | 24 (9%) | 30 (6%) |
| **Other/Unknown** | 26 (19%) | 25 (20%) | 60 (23%) | 111 (21%) |
| **Enrollment Serum Creatinine** |  | | | |
| **median mg/dL (IQR)** | 1**.**2 (0**.**8 – 1**.**9) | 0**.**9 (0**.**7 – 1**.**2) | 0**.**8 (0**.**6 – 1**.**1) | 0**.**9 (0**.**7 – 1**.**2) |
| **Max RIFLE^2^ Stage Within 7 days of Enrollment** |  | | | |
| **No AKI N (%)** | 82 (61%) | 62 (50%) | 126 (48%) | 270 (52%) |
| **RIFLE-R** | 15 (11%) | 32 (26%) | 80 (30%) | 127 (24%) |
| **RIFLE-I** | 7 (5%) | 20 (16%) | 53 (20%) | 80 (15%) |
| **RIFLE-F** | 30 (22%) | 9 (7%) | 6 (2%) | 45 (9%) |
| **Outcomes (within 30 days of enrollment)** |  |  |  |  |
| **RRT N (%)** | 21 (16%) | 3 (2%) | 2 (1%) | 26 (5%) |
| **Death** | 30 (22%) | 16 (13%) | 24 (9%) | 70 (13%) |

**^1^**Percentages for reason for hospital admission do not sum to 100% because more than one reason can be given. **^2^**Original RIFLE criteria as described in the text of the Supplement.

**Table S2. Exposures and Susceptibilities for the Sapphire Study Cohort.**

|  |  | **Endpoint Positive^1^** | **Endpoint Negative^2^** | **Total** | **P Value** |
| --- | --- | --- | --- | --- | --- |
| **All Patients** |  | 101 | 627 | 728 |  |
| **Exposures^3^** |  |  |  |  |  |
|  | **Sepsis** | 26 (26%) | 110 (18%) | 136 (19%) | 0**.**055 |
|  | **Burns** | 1 (1%) | 13 (2%) | 14 (2%) | 0.71 |
|  | **Trauma** | 4 (4%) | 51 (8%) | 55 (8%) | 0.16 |
|  | **Circulatory shock** | 82 (81%) | 521 (83%) | 603 (83%) | 0.67 |
|  | **Major surgery** | 41 (41%) | 252 (40%) | 293 (40%) | 1.0 |
|  | **Radiocontrast agents** | 28 (28%) | 222 (35%) | 250 (34%) | 0.14 |
|  | **Nephrotoxic drugs^4^** | 73 (72%) | 345 (55%) | 418 (57%) | 0.001 |
| **Susceptibilities^5^** |  |  |  |  |  |
|  | **Advanced age (>=75yrs)** | 27 (27%) | 129 (21%) | 156 (21%) | 0.19 |
|  | **Female gender** | 36 (36%) | 243 (39%) | 279 (38%) | 0.58 |
|  | **Black race** | 11 (11%) | 76 (12%) | 87 (12%) | 0.87 |
|  | **CKD** | 14 (14%) | 51 (8%) | 65 (9%) | 0.14 |
|  | **Diabetes mellitus** | 39 (39%) | 171 (27%) | 210 (29%) | 0.064 |
|  | **Cancer** | 25 (25%) | 163 (26%) | 188 (26%) | 0.53 |
|  | **Anemia (HTC<30)** | 42 (42%) | 259 (41%) | 301 (41%) | 1.0 |
|  | **CHF** | 23 (23%) | 99 (16%) | 122 (17%) | 0.17 |
|  | **Liver Disease** | 10 (10%) | 24 (4%) | 34 (5%) | 0.018 |

**^1^**Endpoint positive comprises patients who were KDIGO stage 2 or 3 within 12 hours. **^2^**Endpoint negative comprises patients who were not KDIGO stage 2 or 3 within 12 hours. **^3^** Exposures were defined as follows: (1) Sepsis/Burns/Trauma, listed in hospital record as reason for ICU admission; (2) Circulatory Shock, hypotension (MAP < 70mmHg) within five days prior to and including day of enrollment; (3) Major Surgery, surgery within five days prior to and including day of enrollment and deemed major by a physician; (4) Radiocontrast agents, contrast media (by IV or IA) within five days prior to and including day of enrollment; and (5) Nephrotoxic drugs, any of the following drugs within five days prior to and including day of enrollment: NSAIDs (Ibuprofen, Ketorolac, Naproxen, Celecoxib, Valdecoxib), ACE Inhibitors (Lisinopril, Enalapril, Captopril, Fosinopril), Angiotensin receptor blockers (Losartan, Candesartan, Valsartan), Imunnosuppressants (Cyclosporine, Tacrolimus), Beta Lactam Antibiotics (Ceftriaxone, Cefazolin, Ceftazidime, Piperacillin sodium/Tazobactam sodium), Aminoglycosides (Gentamycin, Tobramycin, Amikacin), Vancomycin, Acyclovir, Amphoteracin/Lypophylized Amphoteracin, Allopurinol, Colistin Sulfate. **^4^**Medications data were missing for one subject. **^5^**Susceptibilities were recorded from the patient’s medical history.

**Table S3. Cox Proportional Hazards Models of [TIMP-2]*•*[IGFBP7] and Serum** Creatinine for the Sapphire cohort.

|  | | | | |
| --- | --- | --- | --- | --- |
| **Model** | **Degrees of Freedom** | **Hazard Ratio For [TIMP-2]***•***[IGFBP7]** | **P-Value^1^** | **C-Statistic (95% CI) ^2^** |
| Serum Creatinine**^3^** | 6 | NA**^6^** | <0**.**0001 | 0.75 (0.70-0.80) |
| [TIMP-2]*•* [IGFBP7]**^4^** | 1 | 6.3 | <0**.**0001 | 0.80 (0.75-0.84) |
| [TIMP-2]*•*  [IGFBP7]  & Serum Creatinine**^5^** | 1 | 4.6 | <0**.**0001 | 0.85 (0.82-0.89) |
| ^1^ Likelihood ratio chi-square test. P-value <0.0001 for the model with [TIMP-2]*•*[IGFBP7] and serum creatinine shows that [TIMP-2]*•*[IGFBP7] adds significant predictive power compared with serum creatinine alone ^2^ Harrell's c-statistic ^3^ Restricted cubic spline transform of Serum Creatinine (used in both models with sCr) ^4^ Log10 transform  the [TIMP-2]*•*[IGFBP7] (used in both models with [TIMP-2]*•*[IGFBP7]). ^5^ [TIMP-2]*•*[IGFBP7] controlling for Serum Creatinine  ^6^ The hazard ratio for serum creatinine is not shown because it changes throughout the range of serum creatinine values, owing to the continuous non-linear shape of the transformation. | | | | |

[TIMP-2]*•*[IGFBP7] and Serum Creatinine were measured in paired urine and serum samples, respectively, and all patients with at least one paired [TIMP-2]*•*[IGFBP7] result and Serum Creatinine value were included in the models (N = 725 patients). The analysis evaluated the relationship between biomarker measurements and the time to the first KDIGO stage 2 or 3 event within 12 hours of biomarker collection, where [TIMP-2]*•*[IGFBP7] and serum creatinine were included as time-dependent covariates. Further details including censoring times and the process for selecting transforms are given in the Statistical Analysis section of the Supplement. The results show that [TIMP-2]*•*[IGFBP7] adds significant predictive power compared with serum creatinine alone.

**Table S4. Cox Proportional Hazards Models For [TIMP-2]*•*[IGFBP7] and Clinical Covariates for the Sapphire Cohort**

|  | **Clinical Model** | | **Clinical Model with**  **[TIMP-2]•[IGFBP7]** | |
| --- | --- | --- | --- | --- |
| **Variable** | **Hazard Ratio** | **p-value^1^** | **Hazard ratio** | **p-value^1^** |
| **Age** | 0.99 | 0.32 | 0.99 | 0.35 |
| **Serum Creatinine^2^** | NA^3^ | <.0001 | NA^3^ | <.0001 |
| **APACHE III Score** | 1.0 | 0.001 | 1.0 | 0.35 |
| **Hypertension** | 2.2 | 0.002 | 2.1 | 0.004 |
| **Nephrotoxic drugs** | 1.6 | 0.033 | 1.4 | 0.12 |
| **Liver Disease** | 2.4 | 0.037 | 2.2 | 0.069 |
| **Sepsis** | 1.4 | 0.18 | 1.3 | 0.32 |
| **Diabetes** | 1.4 | 0.16 | 1.3 | 0.29 |
| **Chronic Kidney Disease** | 0.52 | 0.031 | 0.70 | 0.27 |
| **[TIMP-2]•[IGFBP7]^4^** | Not included in model | | 4.1 | <.0001 |
| **C-statistic (95% CI)^5^** | 0.81 (0.76-0.85) | | 0.87 (0.84-0.90) | |
| ^1^Likelihood ratio chi-square test. P-value <0.0001 for the model with [TIMP-2]*•*[IGFBP7] and clinical covariates shows that [TIMP-2]*•*[IGFBP7] adds significant predictive power compared with the clinical covariates alone.  ^2^Transform for serum creatinine was a restricted cubic spline with 6 degrees of freedom.  ^3^The hazard ratio for serum creatinine is not shown because it changes throughout the range of serum creatinine values, owing to the continuous non-linear shape of the transformation.  ^4^ [TIMP-2]•[IGFBP7] was log transformed.  ^5^ Harrell's c-statistic | | | | |

[TIMP-2]*•*[IGFBP7] and Serum Creatinine were measured in paired urine and serum samples, respectively. All patients with at least one paired [TIMP-2]*•*[IGFBP7] result and Serum Creatinine value and a value for every clinical covariate were included in the models (N = 705 patients). The analysis evaluated the relationship between biomarker measurements and the time to the first KDIGO stage 2 or 3 event within 12 hours of biomarker collection, where [TIMP-2]*•*[IGFBP7] and serum creatinine were included as time-dependent covariates. All other parameters were included as fixed covariates. Note: the clinical model was derived from the entire cohort and thus the C-statistic is likely an overestimate; however this model represents the most stringent test of enhancement by [TIMP-2]*•*[IGFBP7]. All clinical covariates shown in Table 1 of the main manuscript and Table S2 with p-value < 0.1 were included. Chronic kidney disease was also included because it is a known risk factor for AKI. The same results were obtained when chronic kidney disease was omitted from the clinical model (adjusted hazard ratio for [TIMP-2]*•*[IGFBP7] = 4.2 with p-value <0.0001; AUC for the clinical model with [TIMP-2]*•*[IGFBP7] = 0.87 (0.84-0.90)). Further details including censoring times and the process for selecting transforms are given in the Statistical Analysis section of the Supplement. The results show that [TIMP-2]*•*[IGFBP7] remains highly significant after adjustment for clinical covariates.

**Table S5. Generalized Estimating Equation (GEE) Risk Models for [TIMP-2]•[IGFBP7] and Clinical Covariates for the Sapphire Cohort**

|  | **Reference Risk Model** | | **New Risk Model  (addition of [TIMP-2]•[IGFBP7] to reference risk model)** | |
| --- | --- | --- | --- | --- |
| **Variable** | **Odds Ratio** | **p-value** | **Odds Ratio** | **p-value** |
| **Age** | 0.99 (0.98-1.01) | 0.42 | 0.99 (0.97-1.01) | 0.32 |
| **log(serum creatinine)** | 74 (18-315) | <0.0001 | 44 (13-151) | <0.0001 |
| **APACHE III Score** | 1.01 (1.01-1.02) | 0.0003 | 1.01 (1.00-1.02) | 0.067 |
| **Hypertension** | 2.2 (1.2-4.0) | 0.013 | 2.2 (1.2-3.9) | 0.013 |
| **Nephrotoxic drugs** | 1.9 (1.1-3.2) | 0.020 | 1.9 (1.1-3.3) | 0.013 |
| **Liver Disease** | 2.6 (1.2-5.7) | 0.019 | 2.5 (1.0-6.5) | 0.057 |
| **Sepsis** | 1.3 (0.7-2.2) | 0.37 | 1.1 (0.7-2.0) | 0.64 |
| **Diabetes** | 1.5 (0.9-2.4) | 0.12 | 1.3 (0.8-2.1) | 0.35 |
| **Chronic Kidney Disease** | 0.44 (0.22-0.87) | 0.018 | 0.84 (0.40-1.74) | 0.64 |
| **[TIMP-2]•[IGFBP7]** | Not included in model | | 5.0 (3.2-8.0) | <0.0001 |

Models for risk of KDIGO 2 or 3 AKI within 12 hours of sample collection. All patients with at least one paired [TIMP-2]*•*[IGFBP7] and serum creatinine value and values for all clinical variables were included (N=705), All available [TIMP-2]*•*[IGFBP7] and serum creatinine values available within 18h of enrollment were included in a bootstrap analysis. All clinical covariates shown in Table 1 of the main manuscript and Table S2 with p-value < 0.1 were included. Chronic kidney disease was also included because it is a known risk factor for AKI. Further details are described in the Statistical Analysis section of the Supplement.

**Table S6. Integrated Discrimination Improvement (IDI) and Category-Free Net Reclassification Improvement (cfNRI) for [TIMP-2]•[IGFBP7] for the Sapphire Cohort**

| **Statistic** | **Value** | **95% CI** |
| --- | --- | --- |
| **IDI** | 0.098 | (0.032-0.265) |
| **IDI_event** | 0.087 | (0.033-0.326) |
| **IDI_nonevent** | 0.011 | (0.005-0.039) |
| **cfNRI (%)** | 70 | (19-96) |
| **cfNRI_event (%)** | 45 | (8-71) |
| **cfNRI_nonevent (%)** | 25 | (0-43) |
| **AUC_reference_model** | 0.80 | (0.50-0.84) |
| **AUC_new_model** | 0.87 | (0.64-0.90) |
| **AUC_difference** | 0.06 | (0.04-0.36) |

IDI, cfNRI and AUC_difference were calculated based on the reference risk model and new risk model (addition of [TIMP-2]*•*[IGFBP7]) shown in Table S5. Event = KIDGO 2 or 3 AKI within 12h of sample collection. The reference risk model was derived from the entire cohort and thus the AUC is likely an overestimate. However, this therefore represents the most stringent test of enhancement by [TIMP-2]*•*[IGFBP7]. All clinical covariates shown in Table 1 of the main manuscript and Table S2 with p-value < 0.1 were included. Chronic kidney disease was also included because it is a known risk factor for AKI. All [TIMP-2]*•*[IGFBP7] and serum creatinine values available within 18 hours of enrollment were included in a bootstrap analysis, consistent with the primary Sapphire analysis. An analysis using only the first (enrollment) samples yielded similar results (IDI = 0.08; 95% CI = 0.04-0.15 and cfNRI = 68%; 95%CI = 44%-96%). Further details are given in the Statistical Analysis section of the supplement.

| **Urine L-**  **FABP** | **AUC** | 0.61 | 0.58 | 0.60 | 0.57 | 0.63 | 0.61 | 0.59 | **Table S7. Sensitivity analysis of the Sapphire cohort for method of determining the sCr reference value and for exclusion of samples after first occurrence of KDIGO stage 2 or 3.^1^** sCr reference method A: Median of values from six months to six days prior to enrollment (if at least five values available or if more values available than from five days to zero days prior to enrollment) ELSE nadir value from five days to zero days prior to enrollment (if at least one value available) ELSE value closest to enrollment time. sCr reference method B: most recent value from seven days prior to hospital admission (if at least one value) ELSE nadir value from seven days prior to hospital admission to enrollment (if at least one value) ELSE value closest to enrollment. sCr reference method C: most recent value from seven days prior to hospital admission (if at least one value) ELSE excluded from the analysis. A full description of each sCr reference method is given in the text of the Supplement. **^2^**Samples for each patient collected after the patient’s first sample that had a positive KDIGO stage 2 or 3 classification were either excluded from the bootstrap analysis or not, as indicated. A full description is given in the text of the supplement. **^3^**Patients who were properly enrolled but reached KDIGO stage 2 or 3 within 24 hours prior to enrollment (N=39) were either excluded from the analysis or not as indicated. A full description is given in the text of the supplement. For all sensitivity analyses, our conclusions were unchanged and the [TIMP-2]*•*[IGFBP7] AUC was not different from the primary analysis (point estimate for AUC within the 95% confidence interval) and was higher than the AUC of all existing biomarkers. |
| --- | --- | --- | --- | --- | --- | --- | --- | --- | --- |
| **Urine pi-**  **GST** | **AUC** | 0.61 | 0.63 | 0.60 | 0.60 | 0.59 | 0.59 | 0.61 |
| **Urine IL-18** | **AUC** | 0.69 | 0.68 | 0.68 | 0.67 | 0.67 | 0.69 | 0.65 |
| **Plasma NGAL** | **AUC** | 0.69 | 0.68 | 0.68 | 0.67 | 0.69 | 0.70 | 0.69 |
| **Urine KIM-**  **1** | **AUC** | 0.70 | 0.67 | 0.70 | 0.67 | 0.73 | 0.69 | 0.74 |
| **Plasma Cystatin**  **C** | **AUC** | 0.71 | 0.70 | 0.70 | 0.70 | 0.72 | 0.72 | 0.71 |
| **Urine NGAL** | **AUC** | 0.72 | 0.70 | 0.71 | 0.69 | 0.72 | 0.73 | 0.68 |
| **IGFBP7** | **AUC** | 0.76 | 0.75 | 0.77 | 0.75 | 0.77 | 0.76 | 0.75 |
| **TIMP-**  **2** | **AUC** | 0.79 | 0.79 | 0.79 | 0.77 | 0.79 | 0.78 | 0.77 |
| **[TIMP-2]***•*  **[IGFBP7]** | **AUC** | 0.80 | 0.79 | 0.79 | 0.78 | 0.79 | 0.79 | 0.78 |
| **Exclude all but the enrollment sample?** | | **No** | **No** | **No** | **No** | **Yes** | **No** | **No** |
| **Exclude patient if AKI stage 2 or 3 reached before enrollment?^3^** | | **No** | **Yes** | **No** | **Yes** | **No** | **No** | **No** |
| **Exclude samples collected after the first AKI stage 2 or 3 positive sample?^2^** | | **No** | **No** | **Yes** | **Yes** | **No** | **No** | **No** |
| **sCr Reference method^1^** | | **A** | | | | | **B** | **C** |

**Table S8**. Area under the receiver-operating characteristics curve (AUC) for the best novel biomarkers and existing markers analyzed in the Discovery cohorts.

| **Biomarker** | **RIFLE I or F 12-36 hours after sample collection** | | **RIFLE I or F Within 12 hours after sample collection** | |
| --- | --- | --- | --- | --- |
|  | **AUC** | **95% Confidence Interval** | **AUC** | **95% Confidence Interval** |
| **Plasma NGAL** | 0.64 | 0.58 – 0.70 | 0.64 | 0.58 – 0.71 |
| **Urine NGAL** | 0.66 | 0.60 – 0.71 | 0.71 | 0.66 – 0.76 |
| **Plasma Cystatin C** | 0.59 | 0.53 – 0.64 | 0.63 | 0.57 – 0.69 |
| **Urine L-FABP** | 0.54 | 0.48 – 0.60 | 0.66 | 0.60 – 0.72 |
| **Urine IL-18** | 0.65 | 0.60 – 0.71 | 0.76 | 0.71 – 0.81 |
| **Urine KIM-1** | 0.66 | 0.61 – 0.72 | 0.69 | 0.63 – 0.75 |
| **Urine Pi-GST** | 0.61 | 0.55 – 0.67 | 0.65 | 0.60 – 0.71 |
| **Urine TIMP-2** | 0.75 | 0.70 – 0.80 | 0.79 | 0.75 – 0.84 |
| **Urine IGFBP7** | 0.77 | 0.71 – 0.82 | 0.78 | 0.72 – 0.83 |
| **[TIMP-2]***•***[IGFBP7]** | 0.77 | 0.72 – 0.82 | 0.80 | 0.75 – 0.84 |

Data were pooled from all three cohorts (Vienna, Duke, Mayo). The primary methodology for selecting the best novel biomarkers was based on univariate AUC for RIFLE I or F occurring (clinically manifesting) 12-36 hours after sample collection. The best biomarkers were also characterized by AUC for RIFLE I or F within 12 hours after sample collection for samples collected within 18 hours of enrollment, which corresponds to a clinically relevant time period for clinical assessment of critically ill patients entering the ICU. The point estimate of AUC for IGFBP7 is slightly larger than that for TIMP-2 in the former analysis but this is reversed in the latter analysis, whereas the combination of the two biomarkers ([TIMP-2]*•*[IGFBP7]) exhibit the highest point AUC in both analyses. In both analyses, the point estimates of AUC for TIMP-2, IGFBP7, and [TIMP-2]*•*[IGFBP7] are larger than for any existing biomarker. Additional details are given in the text of the Supplement.

**
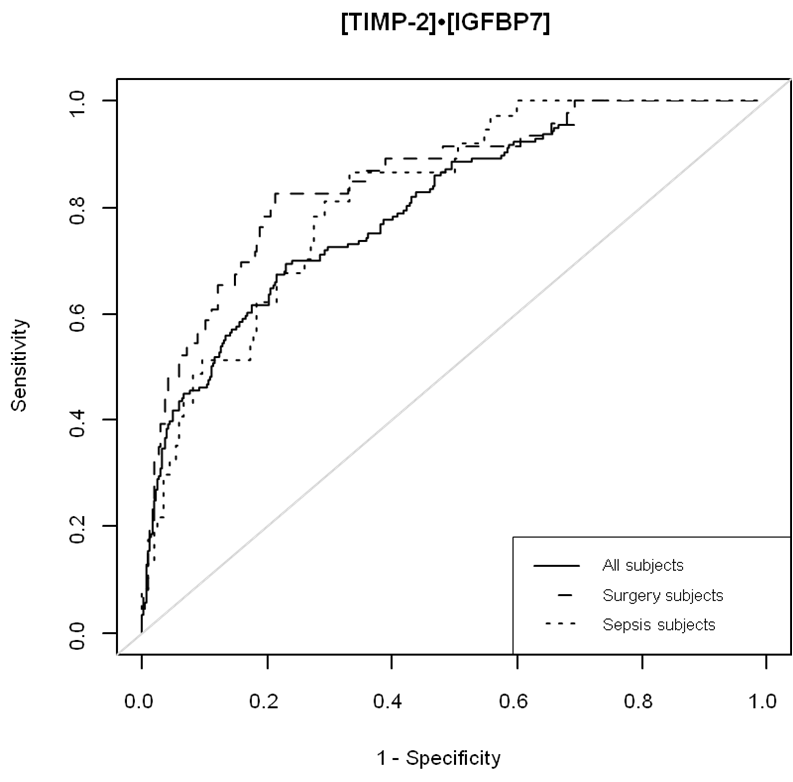
**

**Figure S1.** [TIMP-2]*•*[IGFBP7] Receiver-Operating Characteristics Curves for the Primary Study Endpoint in Sapphire Study Cohorts. The primary study endpoint was KDIGO stage 2 or 3 within 12 hours of sample collection and samples were collected within 18 hours of enrollment, as described in the text of the Supplement. Areas under the curve (AUC) and number of patients were as follows: All subjects, AUC = 0.80, N = 728; surgery subjects (admitted to the ICU because of surgery), AUC = 0.85, N = 247; sepsis subjects (admitted to the ICU for Sepsis), AUC = 0.82, N = 136. Reasons for ICU admission were obtained from the hospital record and some patients (N = 27) were in both the sepsis and surgery cohorts.


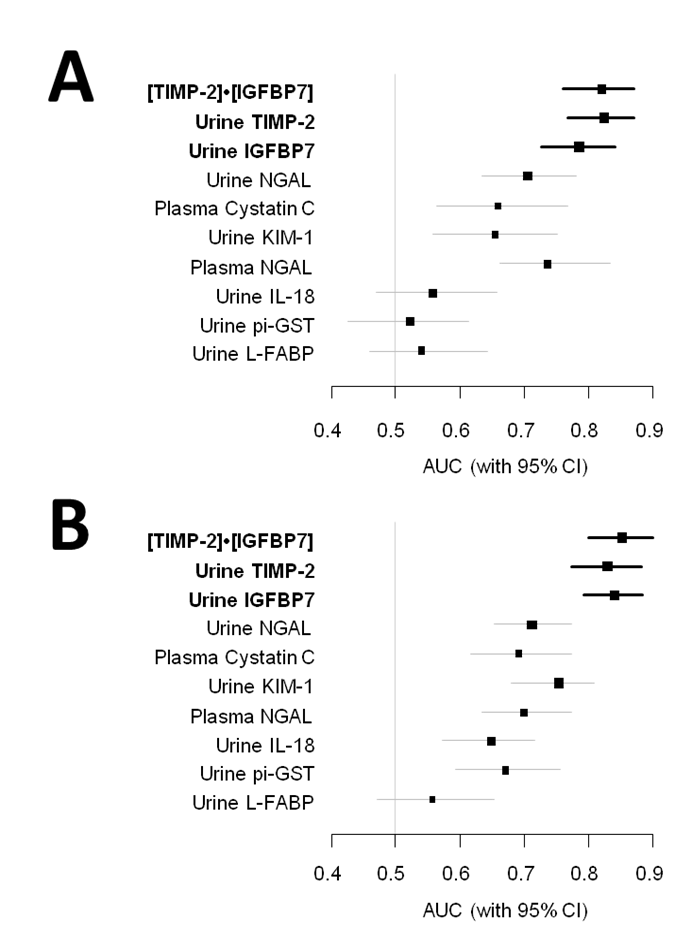


**Figure S2.** Area Under the Receiver-Operating Characteristics Curve (AUC) for the Primary Study Endpoint in Sapphire Study Cohorts. The primary study endpoint was KDIGO stage 2 or 3 within 12 hours of sample collection and samples were collected within 18 hours of enrollment as described in the text of the Supplement. Sapphire cohorts were defined as follows (Number of patients): **(A)** subjects admitted to the ICU for Sepsis (N = 136) and **(B)** subjects admitted to the ICU because of Surgery (N = 247). Reasons for ICU admission were obtained from the hospital record and some patients (N = 27) were in both the sepsis and surgery cohorts. The point estimate of AUC for IGFBP7 is slightly larger than that for TIMP-2 in the surgery cohort but this is reversed in the sepsis cohort, whereas the combination of the two biomarkers ([TIMP-2]*•*[IGFBP7]) exhibits the highest point AUC across cohorts, including for all evaluable patients (shown in the manuscript). In all cohorts, the point estimates of AUC for TIMP-2, IGFBP7, and [TIMP-2]*•*[IGFBP7] are larger than for any existing biomarker.

**
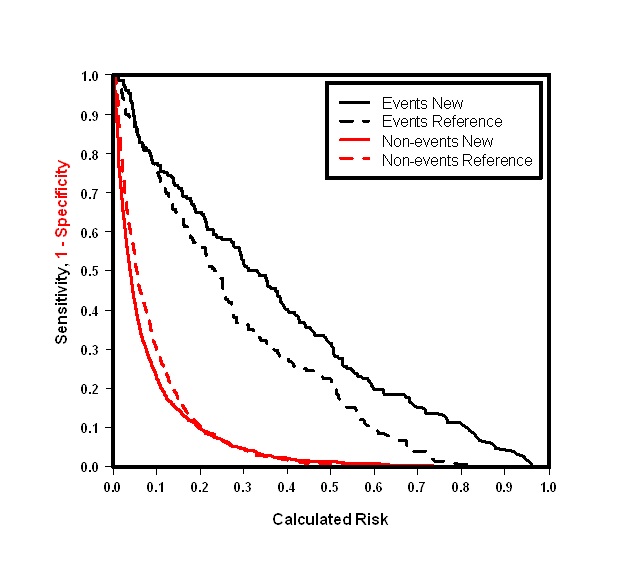
**

**Figure S3.**Clincal model enhancement by adding [TIMP-2]*•*[IGFBP7]. Risk assessment plots for the reference risk model (dashed lines) and new risk models (solid lines) were calculated from the reference and new risk models shown in Table S5. The reference model comprises clinical variables and the new model comprises the clinical variables and [TIMP-2]*•*[IGFBP7]. Red lines represent 1-specificity versus the calculated risk for those without an event (KIDIGO 2 or 3 AKI within 12h); black lines represent sensitivity versus the calculated risk for those with an event. The larger difference between the solid black and red lines (New risk model) compared with the difference between the dashed black and red lines (reference risk model) indicates that addition of [TIMP-2]*•*[IGFBP7] enhances the risk model. Further details are described in the Statistical Analysis section of the Supplement.

**
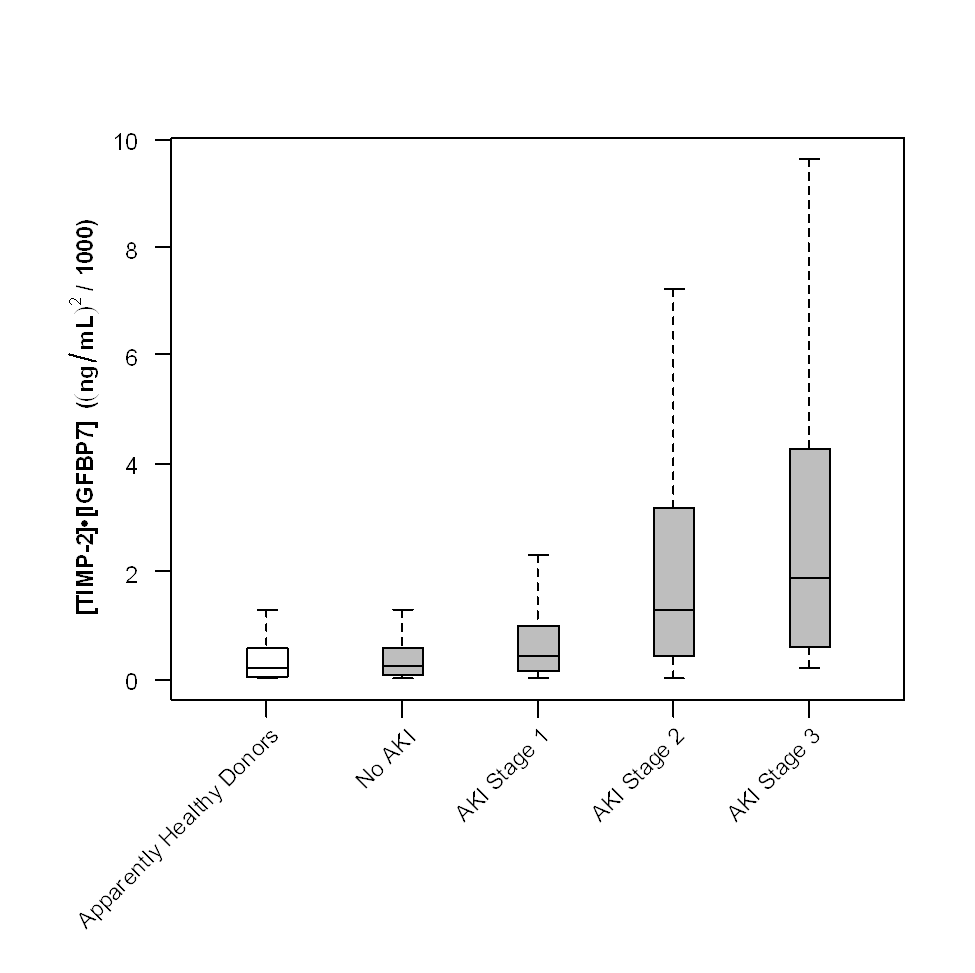
**

**Figure S4.** Box plots showing urine [TIMP-2]•[IGFBP7] values. The open box represents apparently healthy urine donors (N = 383 adult subjects). Shaded boxes represent Sapphire subjects stratified by maximum AKI Stage within 12 hours of sample collection (Number of subjects: no AKI, 416; AKI stage 1, 211; AKI stage 2, 83 and AKI stage 3, 18). Boxes and whiskers show inter-quartile ranges and total observed ranges (censored by 1.5 times the box range), respectively. Sapphire samples were collected within 18 hours of enrollment.

**Supplemental References**

1 Bellomo R, Ronco C, Kellum JA, Mehta RL, Palevsky P: **Acute renal failure - definition, outcome measures, animal models, fluid therapy and information technology needs: The Second International Consensus Conference of the Acute Dialysis Quality Initiative (ADQI) Group.** *Crit Care* 2004, **8**: R204–R212.

2 Kellum JA, Lameire N, Aspelin P, , Barsoum RS, Burdmann EA, Goldstein SL, Herzog CA, Joannidis M, Kribben A, MacLeod AM, Mehta RL, Murray RL, Murray PT, Naicker S, Opal SM, Schaefer F, Schetz M, Uchino S: **KDIGO Clinical Practice Guideline for Acute Kidney Injury 2012.** *Kidney International Supplements* 2012, **2**: 1–138.

3 Siew ED, Matheny ME, Ikizler TA, Lewis JB, Miller RA, Waitman LR, Go AS, Parikh CR, Peterson JF: **Commonly used surrogates for baseline renal function affect the classification and prognosis of acute kidney injury.** *Kidney Int* 2010, **77**: 536–42.

4 Siew ED, Ikizler TA, Matheny ME, Shi Y, Schildcrout JS, Danciu I, Dwyer JP, Srichai M, Hung AM, Smith JP, Peterson JF: **Estimating Baseline Kidney Function in Hospitalized Patients with Impaired Kidney Function.** *Clin J Am Soc Nephrol* 2012, **7**: 712–8.

5 Coca SG, Peixoto AJ, Garg AX, Krumholz HM, Parikh CR: **The prognostic importance of a small acute decrement in kidney function in hospitalized patients: a systematic review and meta-analysis.** *Am J Kidney Dis* 2007, **50**: 712–20.

6 Zavada J, Hoste E, Cartin-Ceba R, Calzavacca P, Gajic O, Clermong G, Bellomo R, Kellum JA, AKI6 Investigators: **A comparison of three methods to estimate baseline creatinine for RIFLE classification.** *Nephrol Dial Transplant* 2010, **25**: 3911–8.

7 Zhou XH, McClish DK, Obuchowski NA: *Statistical Methods in Diagnostic Medicine.* New York: John Wiley & Sons, 2002.

8 Hollander M, Wolfe DA: *Nonparametric Statistical Methods, Second Edition*. New York: John Wiley & Sons, 1999.

9 Lin DY, Wei LJ, Ying Z: **Model-checking techniques based on cumulative residuals.** *Biometrics* 2002, **58**: 1–12.

10 Stone CJ, Koo CY: **Additive Splines in Statistics, In: Proceedings of the Statistical Computing Section ASA**. Washington, DC, 1985: 45–48.

11 Harrell FE: *Regression Modeling Strategies with Applications to Linear Models, Logistic Regression and Survival Analysis.* New York: Springer-Verlag, 2001.

12 Pencina MJ, D'Agostino RB, Sr., Steyerberg EW: **Extensions of net reclassification improvement calculations to measure usefulness of new biomarkers.** *Stat Med* 2011, **30**: 11–21.

13 Parikh CR, Coca SG, Thiessen-Philbrook H, Shlipak MG, Koyner JL, Wang Z, Edelstein CL, Devarajan P, Patel UD, Zappitelli M, Krawczeski CD, Passik CS, Swaminathan M, Garg AX, TRIBE-AKI Consortium: **Postoperative Biomarkers Predict Acute Kidney Injury and Poor Outcomes after Adult Cardiac Surgery.** *J Am Soc Nephrol* 2011, **22**: 1748–1757.

14 Efron B, Tibshirani R: *An Introduction to the Bootstrap, Third Edition*. Boca Roton, FL: Chapman and Hall, 1993.

15 Højsgaard S, Halekoh U, Yan J: **The R Package geepak for GeneralizedEstimating Equations.** *J Stat Softw* 2006, **15**: 1–11.

16 Pickering JW, Endre ZH: **New metrics for assessing diagnostic potential of candidate biomarkers.** *Clin J Am Soc Nephrol* 2012, **7**: 1355–1364.
